# Structure-based redesigning of pentoxifylline analogs against selective phosphodiesterases to modulate sperm functional competence for assisted reproductive technologies

**DOI:** 10.1038/s41598-021-91636-y

**Published:** 2021-06-10

**Authors:** Mutyala Satish, Sandhya Kumari, Waghela Deeksha, Suman Abhishek, Kulhar Nitin, Satish Kumar Adiga, Padmaraj Hegde, Jagadeesh Prasad Dasappa, Guruprasad Kalthur, Eerappa Rajakumara

**Affiliations:** 1 Macromolecular Structural Biology Lab, Department of Biotechnology, Indian Institute of Technology Hyderabad, Kandi, Sangareddy, Telangana 502285 India; 2 Department of Clinical Embryology, Kasturba Medical College, Manipal, Manipal Academy of Higher Education, Manipal, Karnataka 576104 India; 3 Department of Urology, Kasturba Medical College, Manipal, Manipal Academy of Higher Education, Manipal, Karnataka 576104 India; 4 Department of Chemistry, Mangalore University, Mangalagangothri, Karnataka 574199 India

**Keywords:** Biophysics, Drug discovery, Urology

## Abstract

Phosphodiesterase (PDE) inhibitors, such as pentoxifylline (PTX), are used as pharmacological agents to enhance sperm motility in assisted reproductive technology (ART), mainly to aid the selection of viable sperm in asthenozoospermic ejaculates and testicular spermatozoa, prior to intracytoplasmic sperm injection (ICSI). However, PTX is reported to induce premature acrosome reaction (AR) and, exert toxic effects on oocyte function and early embryo development. Additionally, in vitro binding studies as well as computational binding free energy (ΔG_bind_) suggest that PTX exhibits weak binding to sperm PDEs, indicating room for improvement. Aiming to reduce the adverse effects and to enhance the sperm motility, we designed and studied PTX analogues. Using structure-guided in silico approach and by considering the physico-chemical properties of the binding pocket of the PDEs, designed analogues of PTX. In silico assessments indicated that PTX analogues bind more tightly to PDEs and form stable complexes. Particularly, ex vivo evaluation of sperm treated with one of the PTX analogues (PTXm-1), showed comparable beneficial effect at much lower concentration—slower AR, higher DNA integrity and extended longevity of  spermatozoa and  superior embryo quality. PTXm-1 is proposed to be a better pharmacological agent for ART than PTX for sperm function enhancement.

## Introduction

With an estimate of 45.8 million infertile couples worldwide, male infertility accounts for approximately 20–30%^1,2^. It is evident that over the years the quality of human semen has declined remarkably^3,4^. In particular, reduction in sperm motility is one of the major causes of male infertility^5,6^. Motility is essential for the sperm in reaching the site of fertilization during natural conception^7^. In addition, the percentage of motile spermatozoa present in ejaculate helps in deciding the ideal artificial insemination method for infertility treatment^8^. Even though there are no efficient empirical treatments to improve semen quality with respect to increasing motility, sperm motility can be manipulated in vitro. Indeed, identification of factors influencing sperm motility in vitro^9–12^, has immensely benefited assisted conception methods such as intrauterine insemination (IUI), in vitro fertilization (IVF) and intracytoplasmic sperm injection (ICSI). Further, for assisted reproductive technologies (ART), where the frozen-thawed semen and testicular spermatozoa samples are used for infertility treatment, motility enhancement can be beneficial^13^.

The cyclic adenosine monophosphate (cAMP)-dependent protein kinase A (PKA) pathway plays a central role in the complex signaling cascade that underlies sperm motility^14^. cAMP is a key intracellular messenger that regulates physiological functions in spermatozoa such as capacitation, motility, and acrosome reaction, in response to extracellular environment, through activation of cAMP-dependent PKA^15^. In spermatozoa, the level of cAMP is tightly and dynamically regulated by two major enzymes, the adenylyl cyclases (ACs) and the phosphodiesterases (PDEs), which catalyze, respectively, the synthesis and degradation of cAMP^16^.

Presence of mRNA transcripts of PDEs and active PDE10A, PDE1, and PDE4 are reported in human spermatozoa as well as in seminal fluid^17–19^. Activities of spermatozoa are largely dependent on exact nature and localization of the expressed PDEs^6,22–25^. PDE4A and PDE4D have been detected in acrosome of the sperm head, supporting the role of PDE4 in regulation of motility without affecting the acrosome reaction^18^. Furthermore, PDE4 isoforms interact with the A-kinase anchor protein 3 (AKAP3), which tethers PKA and is found in the fibrous sheath of sperm’s flagellum^20^. Localization of PDEs in the acrosomal membrane helps in controlling  the cAMP levels, which has implication in the regulation of sperm chemotaxis during fertilization^18,20–22^. mRNA transcripts of PDE10A isoform were identified in bovine testis as well as in primary spermatocytes and spermatids. In addition, PDE10A enzyme has been reported in the acrosome of the developing spermatids, the mature spermatozoa^23^, and the seminal plasma^19^ contributing to more than 40% of all cAMP-PDE activity in human spermatozoa and up to 50% in the enriched motile sperm fraction^19^. Components of seminal plasma can influence acrosomal function, spermatozoon-zona pellucida binding and also sperm fertilizing ability^24^. In human sperm, PDE1 is found at the equatorial segment of head, mid and principal piece of the flagellum, while PDE3 is present in the post-acrosomal segment of the head^16^.

Both selective and non-selective phosphodiesterase inhibitors (PDEIs) have been used to identify and characterize the roles of PDEs in spermatozoa and to enhance sperm parameters for ART. PDE4 inhibition, using Rolipram, has selectively increased the percentage of motile cells^18,20^. Whereas, ablation of PDE10’s activity by the treatment of sperm with MP-10, an inhibitor specific to PDE10, leads to increase in the sperm phosphotyrosine containing proteins, and this is associated with the acquisition of sperm capacitation and also spontaneous acrosome reaction^19^. Non-selective PDEIs, particularly the methylxanthines such as caffeine, pentoxifylline (PTX), and theophylline are known to stimulate the sperm motility, and also accelerate the capacitation and the spontaneous acrosome reactions^25–28^. PTX is one of the widely used pharmacological agents for aiding viable sperm selection in asthenozoospermic patients and for increasing sperm motility prior to ICSI, but it induces the premature acrosome reaction^29–31^. In addition, it exerts toxic effects on oocyte functioning and also on early embryo development^32^. Since the ability of spermatozoa to fertilize the oocyte lasts for a limited period of time after an acrosome reaction, it is important that this reaction is not initiated until the spermatozoa are in close proximity to the oocyte^33^.

Here we report a rational PTX redesign strategy to enhance sperm function for assisted reproductive technologies (ARTs). Our calorimetry-based binding studies establish that PTX exhibits weak affinity towards sperm-specific PDE enzymes—PDE4A, PDE4D, and PDE10A. Therefore, reversible and covalent inhibitors against these PDEs are designed using a multi-pronged in silico approach consisting of redesigning, docking, and molecular dynamics (MD) simulation. We hypothesized that the redesigned PTX analogues may have better binding affinity towards sperm specific PDEs compared to PTX and could enhance sperm functions, conducive for ART. in silico assessment suggest that PTX analogues are better than parent molecule in binding to PDEs. Further, in an earlier study, ex vivo evaluation of one of the designed PTX analogues on sperm has shown enhanced human sperm motility and longevity compared to the parental PTX molecule with minimum cytotoxicity^34^.

## Results

### Calorimetric binding studies of the PTX and PTXm-1 with sperm specific PDEs

**Figure 1:**
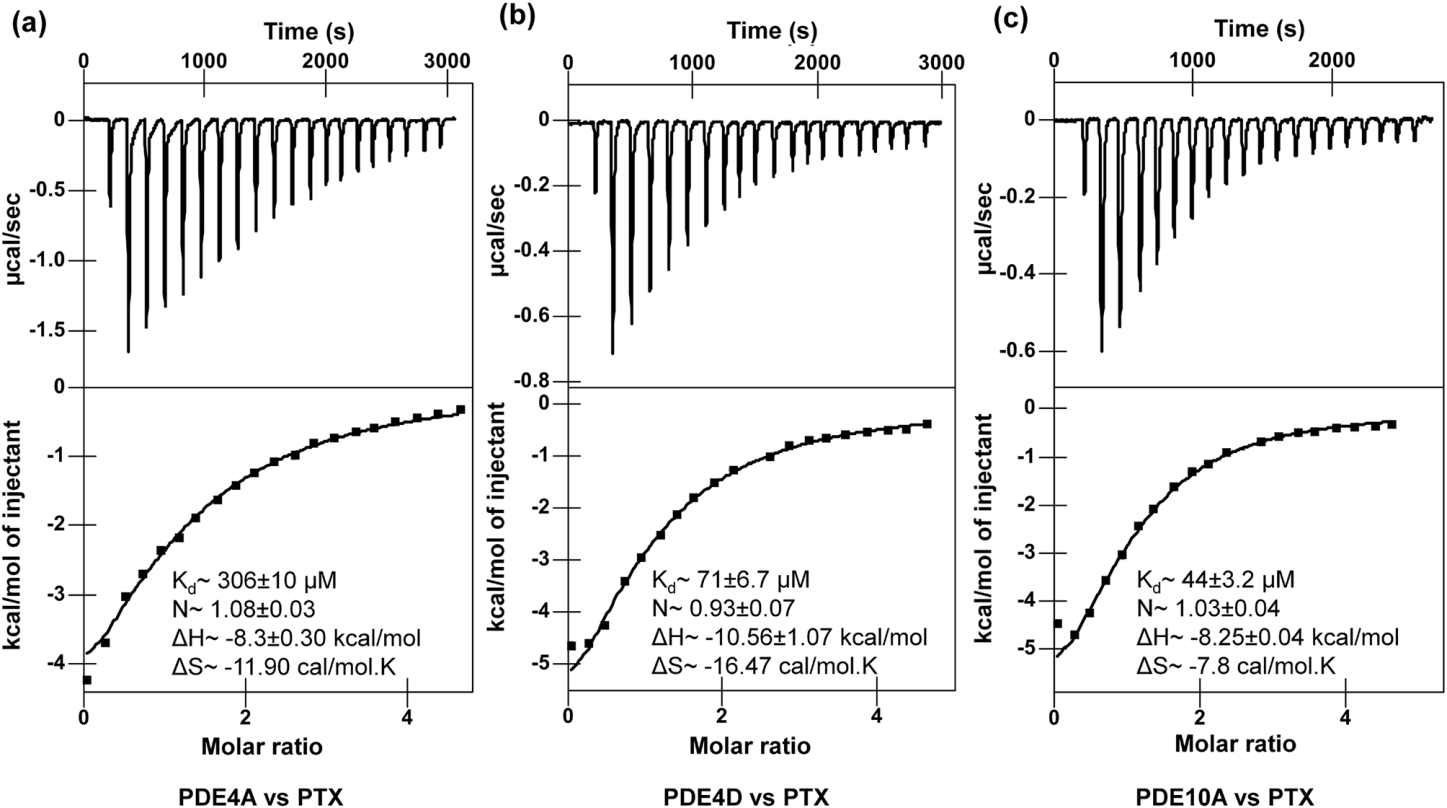
Raw ITC data (upper panel) and normalized integration data (lower panel) for binding of PTX to PDEs. ITC plots for the binding of PTX to (**a**) PDE4A (**b**) PDE4D and (**c**) PDE10A. The inset lists the measured molar dissociation constant (K_d_), stoichiometry (N) and change in enthalpy (ΔH) and entropy (ΔS).

PTX binds to PDE4A, PDE4D and PDE10A with a molar dissociation (K_d_) values of 306, 71, and 44 µM, and corresponding ΔG values of (at T = 298 K) − 4.79, − 5.65 and − 5.93 kcal/mol, respectively. Stoichiometry of binding was found to be one in all the cases (Fig. 1 and Table 1). Unfortunately, poor solubility of PTXm-1 in aqueous solution and precipitation of PDEs upon titrating against PTXm-1 have precluded in vitro isothermal titration calorimetry (ITC) studies. Nevertheless, we have successfully attempted competitive binding studies wherein PTX was titrated against PDE10A-PTXm-1 complex with 2:1 molar ratio. Precipitation of the complex has precluded the use of 1:1 molar ratio of PDE10A-PTXm-1 complex. The PTX binds to PDE10A-PTXm-1 with a K_d_ 350 µM. The presence of PTXm-1 caused ~ eight-fold decrease the binding affinity of PTX towards PDE10A (Supplementary Fig. S1).

**Table 1:** MM-GBSA calculated binding free energy (ΔG_bind_) of interaction of PTX and PTX analogues with PDEs, after 50 ns of MD simulation.

PTX and its analogous	PDE4A	PDE4D	PDE10A
**Reversible binder**			
PTX	− 46.5	− 53.6	− 55.4
	(− 4.79)	(− 5.65)	(− 5.93)
PTXm-1	− **64.3**	− **60.1**	− **69.1**
PTXm-2	− 62.3	− 65.7	− 58.8
PTXm-3	− 35.5	− 59.2	− 31.6
PTXm-4	− **64.8**	− **68.2**	− **68.2**
**Irreversible binder**			
PTXm-5	− **61.5**	− **68.5**	− **61.7**
PTXm-6	− 34.6	− 64.2	− 37.2

Values in parenthesis are ΔG calculated by performing ITC binding studies.

All the values are in kcal.mol^﻿−1^.

### Structure-based in silico design of reversible PTX analogues against the PDEs functioning in the sperm

**Figure 2:**
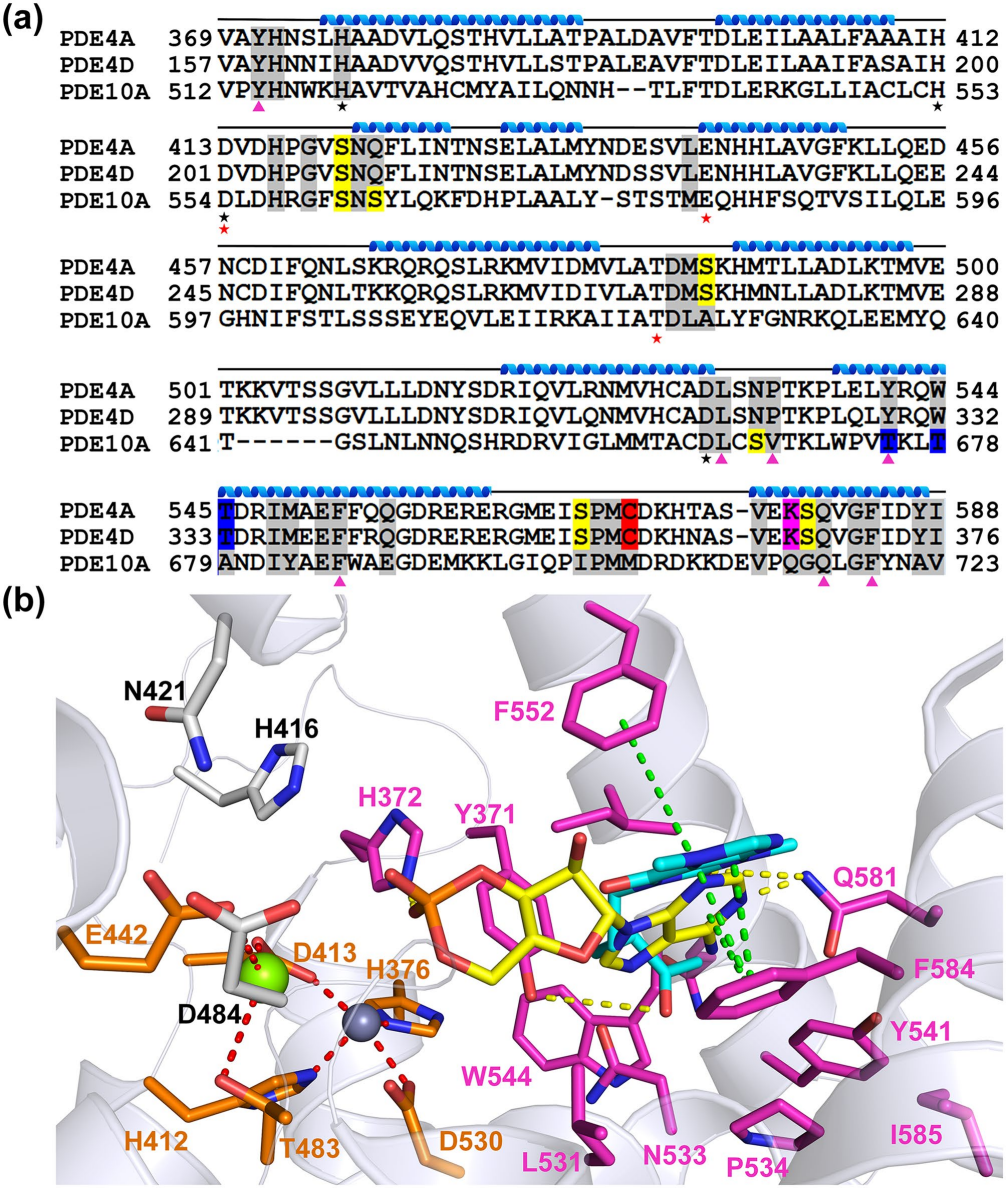
(**a**) Multiple sequence alignment of catalytic domain of PDEs functioning in the sperm. Binding pocket residues are highlighted in different colors. Nucleophilic residues such as Serine (S), Threonine (T), Cysteine (C) and Lysine (K) are highlighted in yellow, blue, red and pink colors respectively. Residues forming coordinated bond with Zn^2+^ and Mg^2+^ ions are represented with black and red star marks, respectively, above the residues. (**b**) PDE4A -PTX binary complex (PDB ID: 3TVX) docked with cAMP. PTX and cAMP are represented in cyan and yellow color sticks, respectively. The metal coordinating residues are represented in orange color sticks. The coordinate bonds between the residues and Zn^2+^ (grey sphere) or Mg^2+^ (green sphere) are shown as the red dashed lines, whereas, the π-stacking interactions between the ligand and the protein residues (magenta stick) and the H-bonds are shown as green and yellow dashed lines, respectively. Other binding pocket residues are represented in grey stick.

Though the cAMP-specific PDEs—PDE4A, PDE4D, and PDE10A—share high sequence and structural similarity (Fig. 2a), variations in the composition of PTX binding residues, which could contribute to the differences in binding affinities (Fig. 1). We used structure of the PDE4A-PTX complex (PDB:3TVX)^35^ as a reference to design PTX analogues to bind PDEs functioning in the sperm with higher affinity. PTX is a tri-substituted xanthine derivative (Supplementary Fig. S2). Sequence and structural analysis showed that the F552 and the F584 of PDE4A, involved in π-stacking interactions with the purine moiety of PTX, are conserved in other PDEs (Fig. 2), implying the importance of purine moiety in designing of PTX analogues. In addition, the polar residues Y371 and N533 (PDE4A numbering), positioned near the ketone group of the aliphatic substituent group of the PTX, are either conserved or positively substituted (Fig. 2). Analysis of interactions of PTX with the binding pocket residues and also the positioning of other non-interacting binding pocket residues (Fig. 2b) were undertaken to design the PTX analogues to enhance the overall interaction network, while avoiding steric clashes, and thus enhancing the stability of the resulting complex.

SiteMap analysis showed that the PTX binding pocket consists of 36 residues, out of which only a few residues are interacting with the PTX (Fig. 2). In addition, volume of the binding pocket in the PDE4A (~ 620 Å^3^) is much bigger than PTX (~ 250 Å^3^) (Fig. 2b), enabling modification of the PTX scaffold with bulkier substituents. Furthermore, the binding pocket has two physico-chemically complementary regions: one predominantly hydrophobic with two polar residues (Q581, Y541), and another predominantly polar with two hydrophobic residues (F552, Y371). These connected regions are ideal for binding the fused aromatic rings with a polar substituent, and also a purine moiety (Fig. 2). Modifications to PTX, sought on basis of the above analyses, included: (A) change in the length of the PTX’s aliphatic substituent (PTXm-4) (B) attaching a 2-methoxy-6-vinylnaphthalene (PTXm-1, PTXm-2), and (C) removing the purine moiety entirely (PTXm-3) (Supplementary Fig. S2).

### Docking and MD simulation analysis of designed analogues

Docked PTX orientation in the binding pockets of PDE4D and PDE10A is similar to its orientation in the crystal structure of PDE4A-PTX complex (PDB ID: 3TVX)^35^ (Supplementary Fig. S3). The designed PTX analogues also bind to the same binding pocket. To check the stability of the docked PTX and the designed analogues in the binding pocket of PDEs, we performed a MD simulation on each complex and computed the free energy of binding (ΔG_bind_). Insignificant fluctuations in the profiles of root mean square deviation (RMSD) and root mean square fluctuation (RMSF), during the MD simulation studies suggest that the PTX and its analogues remained stable in the binding pocket (Supplementary Figs. S4 and S5). PTXm-1 and PTXm-4 have exhibited significantly higher ΔG_bind_ for PDE4A, PDE4D and PDE10A compared to other reversible analogues (Table 1).

### Structural analysis of PTXm-1 binding to PDEs

**Figure 3:**
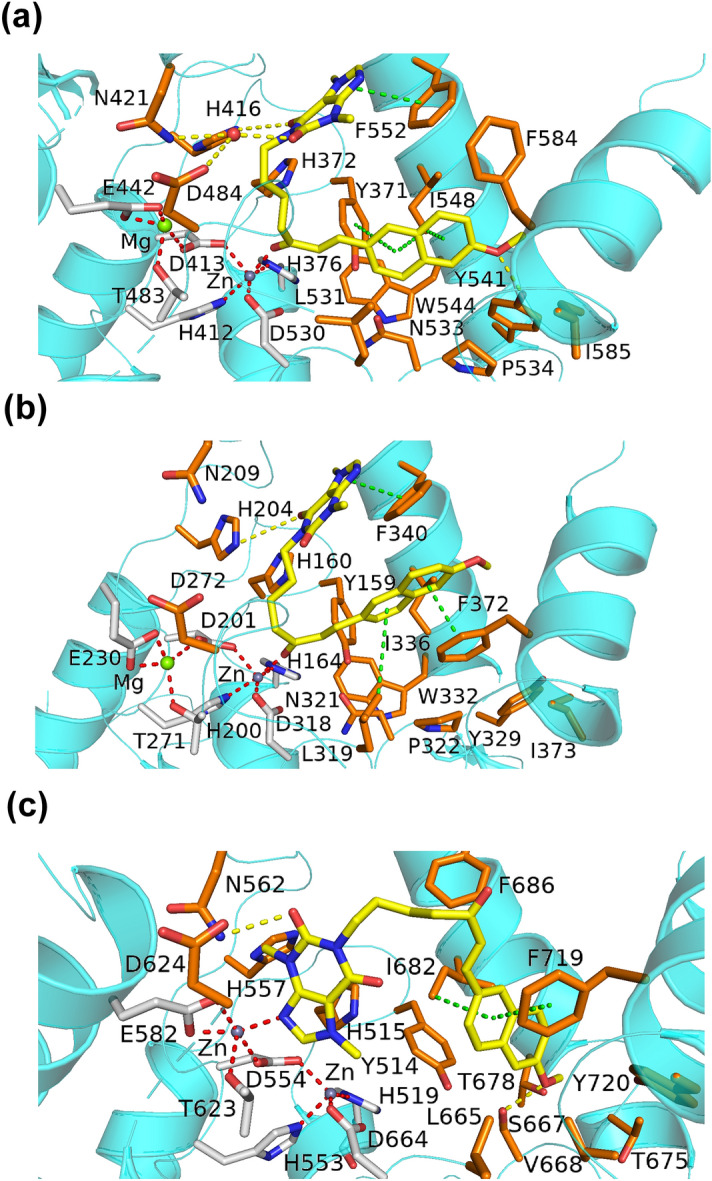
Interaction of PTXm-1 with (**a**) PDE4A (**b**) PDE4D and (**c**) PDE10A. PTXm-1 is shown in yellow sticks, and water, magnesium ion and zinc ion were shown as red, green and grey spheres respectively.

Docking and MD simulation studies have shown that PTXm-1 binds to the PTX binding site, but in an alternate orientation (Figs. 2B and 3), as we predicted. In PTXm-1 purine and naphthalene rings are at the opposite terminals of the aliphatic chain, whereas in PTX, there is only purine ring. The other terminal of the aliphatic chain of PTX has a ketone group (Supplementary Fig. S2). As per our prediction, upon docking, the naphthalene structure was found to bind to the hydrophobic pocket formed by Y371, L531, Y541, W544, and F584 in PDE4A and to residues at equivalent positions in PDE4D and PDE10A, through π-stacking and hydrophobic interactions. In contrast, this hydrophobic region was occupied by the purine part of the PTX in the PDE4A-PTX complex structure (PDB ID: 3TVX), which may have destabilizing effect on PTX binding. The purine ring in PTXm-1 was stabilized by a pocket formed by F552, N421 and H416 residues in the PDE4A. In addition, it has network of π-stacking interaction with F552, H-bond with H416 and a stable water bridge interaction with N421 in PDE4A, throughout the MD simulation (Fig. 3). This region was occupied by solvent in the PDE4A-PTX complex structure, and that could destabilize the PTX binding to the PDEs. Low RMSD of the PDEs and the PTXm-1 in the binding pocket suggest PTXm-1 could form a stable complex with the PDEs through binding in the catalytic pocket of PDEs. Whereas, low RMSF of PTXm-1 indicates the stability of the ligand conformation in the binding pocket (Supplementary Fig. S5). PTXm-1 (~ 400 Å^3^), being a bigger molecule than PTX, but lesser than binding pocket volume, occupies more of the available space in the PDEs’ binding pocket. Our strategy of having two fused ring structures and the increased length of aliphatic chain in PTXm-1 (Supplementary Fig. S2) has allowed it to reach out the deeper in the binding pocket cavity which was unexplored by PTX. These changes are also helpful for PTXm-1 to interact with metal ions present in the catalytic site of PDEs (Fig. 3 and Supplementary Fig. S3). These modifications played an important role in stabilizing the PTXm-1 in the binding pocket of PDEs via π-stacking interactions with aromatic and hydrophobic residues, which further reinforce the stable binding as reflected by lesser ΔG_bind_ (Table 1) compared to PTX. Another PTX analogue, PTXm-4, also showed better binding than PTX as indicated by the RMSD profile of PDEs-PTXm-4 complex and the RMSF of the ligand in the PDEs’ binding pocket and also lower ΔG_bind_ (Supplementary Fig. S6).

### Docking and MD simulation studies of designed covalent inhibitor

The most robust approach to design irreversible inhibitors is to capitalize on the nucleophillic residues, especially cysteine and serine, in the ligand or substrate or co-factor binding pocket of the receptor or the enzyme. Sequence alignment (Fig. 2a) and structural comparison was used to map the position of nucleophilic residues in the substrate/PTX binding pocket of the different PDEs (Supplementary Fig. S7). The position of the nucleophilic residues is highly conserved in the PDE4A and the PDE4D, but those of PDE10A’s are divergent (Fig. 2a and Supplementary Fig. S7). Based on docking and MD simulations, PTXm-1 was identified as a potential scaffold to design the PTXm-5, a covalent inhibitor, PTXm-5 has an acrylamide (warhead group) covalently linked to ‘C8’ of xanthine structure in the PTXm-1 (Supplementary Fig. S2), which can form a covalent bond with nucleophilic residues such as cysteine and serine via Michael addition reaction.

**Figure 4:**
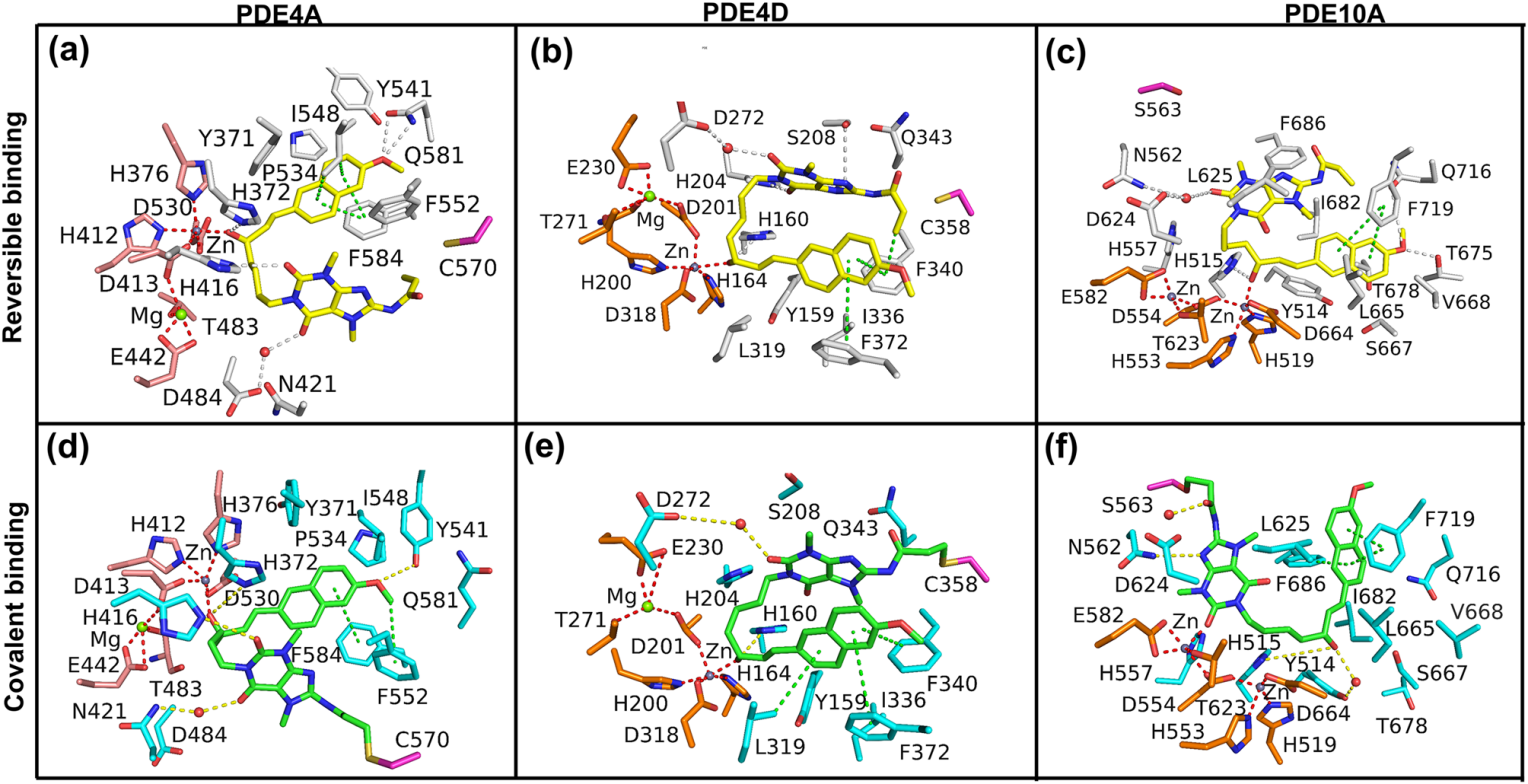
Interactions of pentoxifylline derivative PTXm-5 with PDEs at the end of MD simulations. Reversibly bound PTXm-5 (yellow stick) with (**a**) PDE4A, (**b**) PDE4D and (**c**) PDE10A. Target amino acid for covalent docking is shown as magenta stick. PTXm-5 (green) covalently bound to (**d**) C570 in PDE4A, (**e**) C358 in PDE4D and (**f**) S563 in PDE10A.

First of all, reversible docking of PTXm-5 to PDE4A, PDE4D and PDE10A followed by MD simulation of the complexes was performed to explore the effect of the warhead group on the binding conformation, and the orientation of the ligand in the binding pocket compared to PTXm-1. The results showed that PTXm-5 forms a stable complex with all the PDEs (Fig. 4a–c and Supplementary Fig. S8), and establishes a network of interactions with PDEs similar to PTXm-1 (Fig. 3a–c). The warhead group of PTXm-5 is at a distance of ~ 4 Å from the C570 in the PDE4A and a distance of ~ 3.5 Å from C358 in PDE4D, the nucleophilic residues in the binding pocket of respective proteins (Fig. 4a,b). However, in PDE10A, S563 was the closest nucleophilic residue to PTXm-5 (Fig. 4c), as the equivalent position of C358 of PDE4A is occupied by the methionine in the PDE10A (Fig. 2a and Supplementary Fig. S7). Therefore, C570 in PDE4A, C358 in PDE4D and S563 in PDE10A are targeted for covalent binding with the warhead group of PTXm-5. The covalent docking of PTXm-5 to PDE4A and to PDE4D also show the similar orientation of PTXm-5 in the binding pocket as that of reversible docking (Fig. 4). Though PTXm-5 bound to PDE10A with a slight difference in its orientation during the reversible and the irreversible binding, the network of interactions are conserved (Fig. 4c,f). Insignificant fluctuations in RMSD of the proteins and the ligand, and also negligible change in the RMSF profiles of ligand in the complexes suggest that covalent PTXm-5-PDE complexes are stable throughout the MD simulation (Supplementary Fig. S9).

### Effect of PTXm-1 on sperm motility in ejaculated spermatozoa

**Figure 5:**
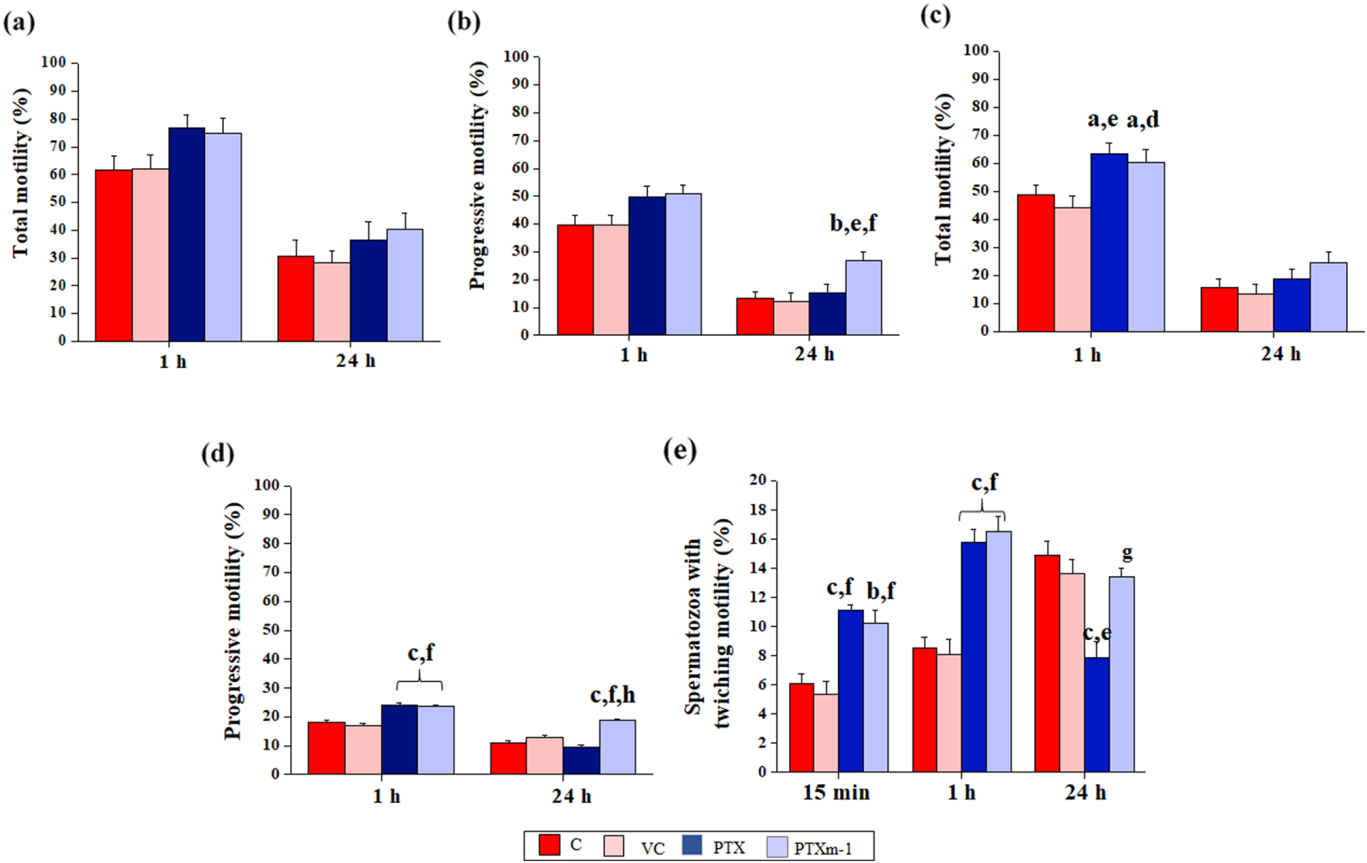
Effect of PTX (1 mM) and PTXm-1 (0.25 mM) on the sperm motility in the normozoospermic and the asthenozoospermic samples processed by swim-up technique at 1 and 24 h intervals. Percentage of (**a**) total and (**b**) progressively motile spermatozoa in the normozoospermic semen samples (N = 84). Percentage of (**c**) total and (**d**) progressively motile spermatozoa in the asthenozoospermic semen samples (N = 28). (**e**) Twitching motility of the testicular spermatozoa (N = 8). The error bar represents standard error to mean. a: *P* < 0.05; b: *P* < 0.01, c: *P* < 0.001 compared to C (control); d: *P* < 0.05, e: *P* < 0.01, f: *P* < 0.001 compared to VC (vehicle control); g: *P* <  < 0.01, h: < 0.001 compared to PTX. C: control; VC: vehicle control; PTX: pentoxifylline; PTXm-1: modified pentoxifylline.

Both PTX and PTXm-1 treatments increased the percentage of total motile spermatozoa in the normozoospermic samples (Table 2) at 1 h and 24 h time intervals compared to controls (Fig. 5a). However, no significant difference in the sperm motility enhancement was observed between PTX and PTXm-1. Decrease in motile spermatozoa at 24 h interval in all the groups indicated the time-dependent decrease in survival. In contrast, higher percentage of progressively motile spermatozoa was observed in PTXm-1 group (*P* < 0.01 compared to C and VC; *P* < 0.05 compared to PTX) at 24 h interval, suggesting that PTXm-1 is more effective in extending the longevity of spermatozoa compared to PTX and control (Fig. 5b).

**Table 2:** Semen characteristics of subfertile men.

Parameters	Normozoospermia (N = 84, Mean ± SE)	Asthenozoospermia (N = 28, Mean ± SE)
Age (Years)	33.42 ± 2.45	38.79 ± 3.81
Sperm conc. (millions/mL)	58.46 ± 3.61	29.78 ± 4.63
Total motility (%)	51.67 ± 3.52	37.45 ± 2.53
Progressive motility (%)	36.45 ± 2.64	14.67 ± 1.63
Normal morphology (%)	31.64 ± 5.52	19.62 ± 1.83
Viability (%)	63.72 ± 3.64	46.49 ± 2.04

Similarly, in asthenozoospermic samples (Table 2), total and progressive motility was significantly higher in PTX and PTXm-1 groups (*P* < 0.001) at 1 h interval compared to controls (Fig. 5c,d). Among PTX and PTXm-1 groups, PTXm-1 had considerably (*P* < 0.001) higher percentage of total and progressively motile spermatozoa at 24 h interval inferring a better  in vitro  sperm survival in this group as a correlation between sperm motility and survival.

### Effect of PTXm-1 on sperm motility in testicular spermatozoa

When the testicular spermatozoa were observed under the microscope, at 15 min after incubation, the control group had 5.06 ± 0.98% of spermatozoa with twitching motility which was similar in vehicle control group (Fig. 5e). Significantly higher percentage of spermatozoa with twitching motility was observed in PTX (11.78 ± 1.36%, *P* < 0.001) and PTXm-1 (9.95 ± 0.79%, *P* < 0.01) compared to control. Increase in the incubation time resulted in increase in the number of motile spermatozoa in all the groups. As observed at 15 min interval, the number of spermatozoa with twitching motility were found to be significantly higher (*P* < 0.001) in both PTX and PTXm-1 (Fig. 5e). However, upon prolonged incubation up to 24 h, the motility was increased in control and vehicle control group; marginally decreased in PTXm-1 group (13.39 ± 0.65%) and significantly decreased (*P* < 0.05) in PTX group (7.90 ± 1.06%) when compared to the motility in the respective groups at 1 h (Fig. 5e). This suggests that PTXm-1 has a superior motility inducing effect on testicular spermatozoa compared to PTX.

### Effect of PTXm-1 on acrosome reaction and DNA integrity of sperm

**Figure 6:**
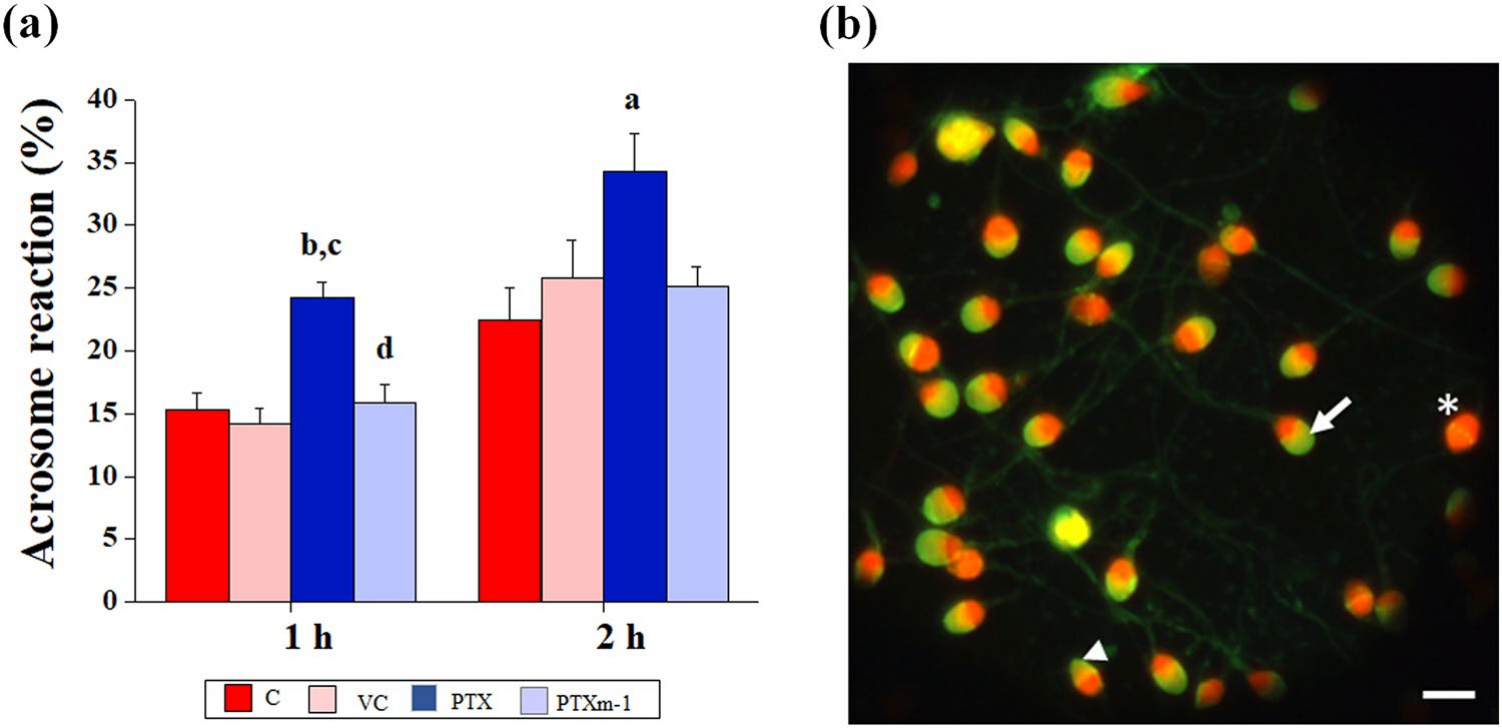
Effect of PTX and PTXm-1 in sperm wash media on the ability of spermatozoa to undergo acrosome reaction in normozoospermic samples assessed by calcium ionophore-induced acrosome reaction assay. (a) percentage of acrosome reacted spermatozoa at 1 h and 2 h after swim up (N = 28). (**b**) Representative image showing spermatozoa with intact acrosome (arrow), spermatozoa after partial acrosome reaction (arrow head) and acrosome reacted spermatozoa (asterix). Spermatozoa were stained with FITC (Fluoresciene isothiocynate) -conjugated *Pisum sativum* agglutinin and counterstained with propidium iodide. The error bar represents standard error to mean. a: *P* < 0.01, b: *P* < 0.001 compared to control; c: *P* < 0.001 compared to vehicle control; d: *P* < 0.001 compared to PTX at respective time. Magnification 400x; scale bar represents 10 µm. C: control; VC: vehicle control; PTX: pentoxifylline; PTXm-1: modified pentoxifylline.

**Figure 7:**
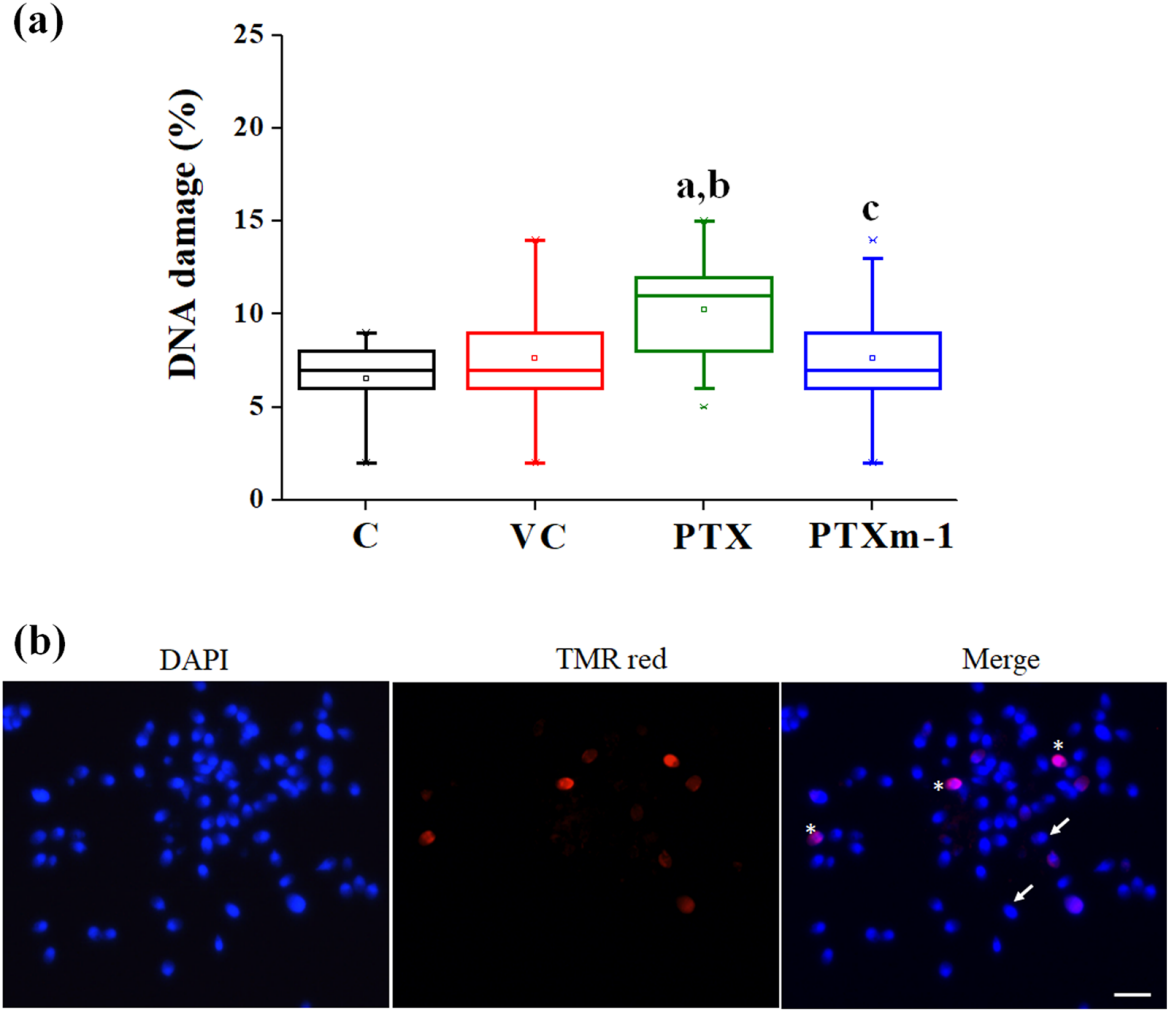
Effect of PTX and PTXm-1 in sperm wash media on DNA integrity of spermatozoa from normozoospermic samples. (**a**) The box and whisker plot represents median percentage of DNA integrity assessed by TUNEL assay at 24 h after swim up (N = 28). The error bar represents standard error to mean. (**b**) Representative image showing spermatozoa with intact DNA (arrow) and with DNA damage (TMR red positive, asterix). a: *P* < 0.001 compared to control; b: *P* < 0.01 compared to vehicle control; c: *P* < 0.01 compared to PTX. Magnification 400x; scale bar represents 10 µm. C: control; VC: vehicle control; PTX: pentoxifylline; PTXm-1: modified pentoxifylline.

We assessed the calcium ionophore-induced acrosome reaction (AR) at 1 and 2 h of incubation with PTX and PTXm-1 in the normozoospermic samples. Surprisingly, AR of spermatozoa was significantly (*P* < 0.001) higher in PTX (24.25%, 95% CI 12.47–17.95) not only compared to control (15.21%, 95% CI 12.47–17.95) but also to PTXm-1 (15.78%, 95% CI 12.88 -18.68) in 1 h the time intervals (Fig. 6a,b). At 2 h interval, PTX has higher percentage (34.28%, 95% CI 26.14–40.47) of acrosome-reacted spermatozoa compared to PTXm-1 (25.17%, 95% CI 22.09–28.187). In addition, at 24 h after in vitro incubation, the spermatozoa from PTX group (10.25%, 95% CI 9.15–11.35) had considerably higher percentage of spermatozoa with damaged DNA (Fig. 7a,b) compared to control (6.5%, 95% CI 5.79–7.20, *P* < 0.001), vehicle control (7.54%, 95% CI 6.36–8.91, *P* < 0.01) and PTXm-1 ( 7.64%, 95% CI 6.38–8.91, *P* < 0.01) indicating lesser DNA damage by PTXm-1.

### Kinematics in spermatozoa

When the kinematics in asthenozoospermic semen samples was assessed at 1 h interval, significant increase in VCL (*P* < 0.001 and *P* < 0.01 in PTX and PTXm-1, respectively), ALH (*P* < 0.001), STR (*P* < 0.001) and WOB (*P* < 0.001) was observed (Table 3). BCF was altered only by PTXm-1 which was significantly higher than control, vehicle control as well as PTX (*P* < 0.01). However, no changes were observed in VSL, LIN and VAP in either PTX or PTXm-1 group.

**Table 3:** Effect of PTX and PTXm-1 on sperm kinematics at 1 h after incubation using computer assisted semen analysis (CASA) in asthenozoospermic semen samples.

Parameters	C	VC	PTX	PTXm-1
VCL (µm/s)	94.50 ± 2.80	96.50 ± 3.24	112.60 ± 3.10^b^	109.6 ± 3.27^a^
VSL (µm/s)	27.54 ± 3.89	26.03 ± 3.56	28.34 ± 4.67	27.67 ± 3.79
ALH (µm)	3.60 ± 0.07	4.50 ± 0.06	5.04 ± 0.06^b^	5.21 ± 0.09^b^
VAP (µm/s)	46.78 ± 0.78	44.78 ± 0.89	47.89 ± 0.96	45.90 ± 0.97
STR	58.87 ± 0.95	58.12 ± 1.00	66.94 ± 0.98^b,c^	67.53 ± 0. 87^b,c^
LIN	29.14 ± 0.98	26.97 ± 0.79	28.47 ± 1.01	27.78 ± 1.02
WOB	49.50 ± 0.73	46.40 ± 0.56	42.54 ± 0.61^b^	41.87 ± 0.70^b^
BCF (Hz)	8.57 ± 0.19	7.98 ± 0.16	8.56 ± 0.17	9.67 ± 0.19^b,c,d^

The error value represents standard error to mean, N = 28).

C, control; VC, vehicle control; PTX, pentoxifylline; PTXm-1, modified pentoxifylline.

^a^*P* < 0.01, ^b^*P* < 0.001 compared to control; ^c^*P* < 0.001 compared to vehicle control; ^d^*P* < 0.001 compared to PTX.

### cAMP level and protein tyrosine phosphorylation in spermatozoa

**Figure 8:**
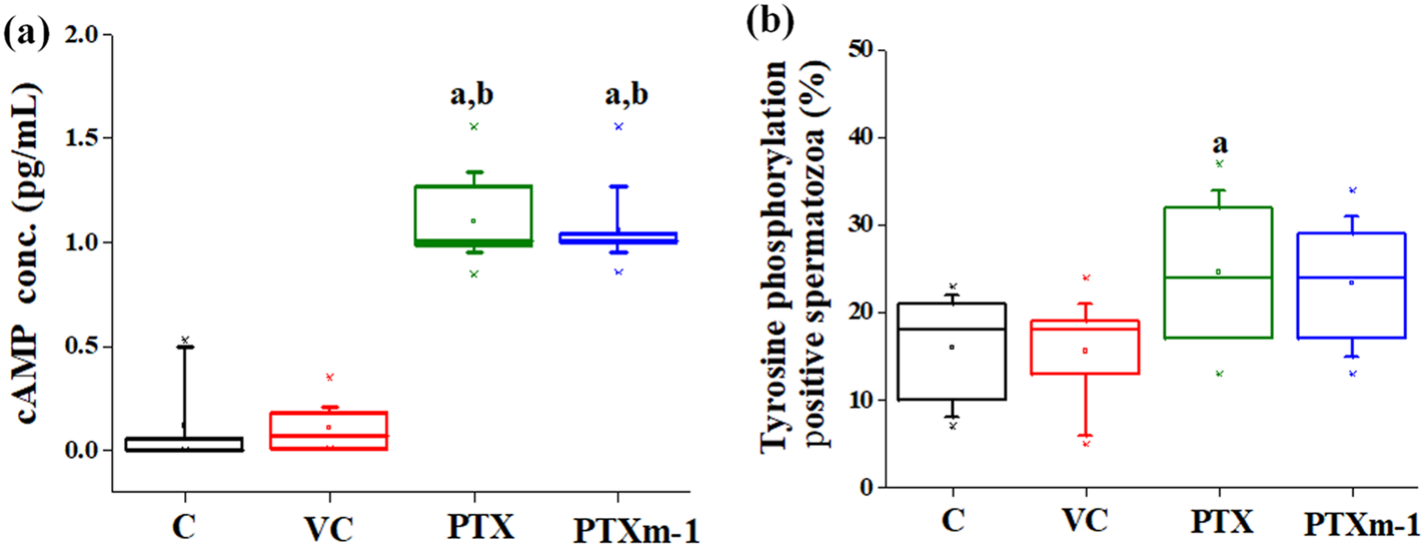
Effect of PTX and PTXm-1 on intracellular cAMP and protein tyrosine phosphorylation levels. (**a**) The box and whisker plot represents median percentage of intracellular cAMP concentration in spermatozoa (N = 8). The error bar represents standard error to mean. a: *P* < 0.001 compared to control; b: *P* < 0.001 compared to vehicle control. (**b**) The box and whisker plot represents median percentage of spermatozoa that underwent protein tyrosine phosphorylation (N = 8). The error bar represents standard error to mean. a: *P* < 0.05, b: *P* < 0.01 compared to control; c: *P* < 0.05, d: *P* < 0.01 compared to vehicle control. C: control; VC: vehicle control; PTX: pentoxifylline; PTXm-1: modified pentoxifylline.

The intracellular cAMP level measured at 1 h after incubation was significantly higher in both PTX and PTXm-1 group compared to control and vehicle control (*P* < 0.001) (Fig. 8a). Assessment of tyrosine phosphorylation of sperm proteins by immunofluorescence indicated that percentage of spermatozoa with tyrosine phosphorylated proteins were significantly higher in PTX (24.75%, 95% CI 19.92–29.30, *P* < 0.01) and in PTXm-1 (23.25%, 95% CI 19.30–27.46, *P* < 0.05) group compared to control (16%, 95% CI, 12.59–19.14) and vehicle control (15.61%, 95% CI 12.12–19.11) (Fig. 8b). Therefore, no significant difference in cAMP or the sperm protein phosphorylation level was observed between PTX and PTXm-1.

### Effect of PTXm-1 supplementation on murine IVF and pre-implantation embryo development

**Figure 9:**
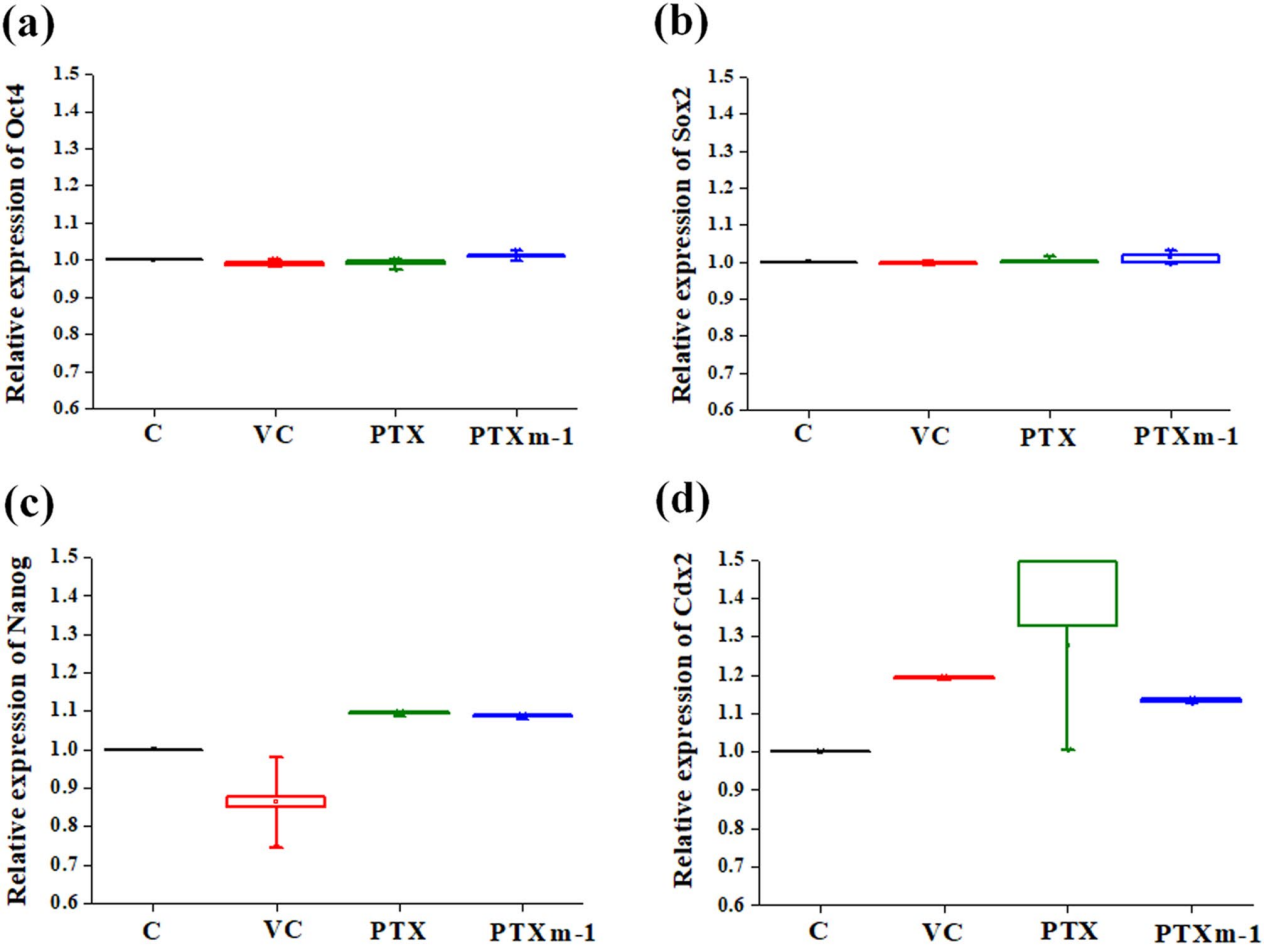
Effect of PTX and PTXm-1 on mRNA expression of pluripotency and trophectoderm genes in blastocysts derived from in vitro fertilized embryos from Swiss albino mice. The box and whisker plot represents median relative expression of pluripotency genes (**a**) Oct4, (**b**) Sox2, (**c**) Nanog, and trophectoderm gene (**d**) Cdx2. The error bar represents standard error to mean (N = 30 embryos each, 2 trials). C: control; VC: vehicle control; PTX: pentoxifylline; PTXm-1: modified pentoxifylline.

At 16 h post insemination, fertilization rate was significantly higher (*P* < 0.01) in both PTX (95.64%) and PTXm-1 (96.25%) groups compared to control and vehicle control (Table 4). Further, in both these groups higher percentage of zygotes progressed to blastocyst stage (*P* < 0.05), indicating that both PTX and PTXm-1 improved fertilization and did not affect the embryo development in vitro (Table 4). No significant difference was observed with respect to the hatching potential of the blastocysts among all the groups (Table 4). However, the blastocysts from PTX group has significantly higher apoptotic index (Table 4) compared to control, vehicle control and PTXm-1 group (*P* < 0.01) as indicated by DNA damage. The total cell number in blastocysts and expression of pluripotency genes did not differ between the groups (Table 4 and Fig. 9).

**Table 4:** Effect of PTX and PTXm-1 on the in vitro fertilization and embryo development potential in Swiss albino mice.

Parameters	C	VC	PTX	PTXm-1
No. of oocytes inseminated	180	168	210	198
Fertilization rate at 16 h post insemination (%)	83.56	85.37	95.64^b,c^	96.25^b,c^
Blastocyst rate at 120 h post insemination (%)	81.67	80.69	86.34^a,c^	88.45^b,d^
Hatching rate at 120 h post insemination (%)	64.56	63.01	67.56^a,c^	66.89^b,d^
TUNEL index (Mean ± SE)	7.25 ± 0.21	7.38 ± 0.32	9.20 ± 0.30	7.40 ± 0.32
Total cell number (Mean ± SE)	92.78 ± 6.58	93.56 ± 7.45	88.91 ± 6.56^b,c^	93.23 ± 8.56^e^

The error value represents standard error to mean.

C, control; VC, vehicle control; PTX, pentoxifylline; PTXm-1, modified pentoxifylline.

^a^*P* < 0.05, ^b^*P* < 0.01 compared to control; ^c^*P* < 0.05, ^d^*P* < 0.01 compared to vehicle control; ^e^*P* < 0.05 compared to PTX.

## Discussion

Comparison reveals that PTX binds to PDE functioning in the sperm with an increased affinity in the order of PDE4A, PDE4D and PDE10A (Fig. 1). Direct correlation between experimental (ITC) ΔG of binding and computational ΔG_bind_ for PTX binding to PDE10A, PDE4D and PDE4A (Table 1) suggest that computational ΔG_bind_ is also correlated with binding affinity (Fig. 1). Therefore, we have used computational ΔG_bind_ of PTX's derivatives as the indicator of strength of binding and for a comparison of PTX with that of its derivatives vis-à-vis binding affinity to above PDEs (Table 1)_._ The presence of PTXm-1 caused ~ eight-fold decrease in the binding affinity of PTX towards PDE10A, indicating that PTXm-1 may bind in the same or overlapping site as PTX (Supplementary Fig. S1). Finally from weak binding affinity of PTX to PDEs we infer that PTX could not be a potent inhibitor of these enzymes. Therefore, with a view to designing a better inhibitor against PDEs, we designed PTX analogues and performed in silico screening using docking and MD simulation studies. Crystal structure of PDE4A-PTX complex^35^ showed that there are two prominent regions in the binding pocket: one which is rich in aromatic residues and the other one is rich in polar residues (Fig. 2a). Based on this and the surplus volume of binding pocket we have used a strategy of fusing an aliphatic end of the PTX with a naphthalene ring, surmising that a naphthalene ring and a purine ring could bind to the hydrophobic rich and the polar rich regions, respectively. We have also optimized the linker region between the two rings such that both ring structures should optimally bind to corresponding regions in the binding pocket. Docking, MD simulation and computational ΔG_bind_ analyses of PTX analogues and a comparison with parent PTX molecule indicated that redesigned PDEIs are more potent than PTX.

The designed PTX analogues exhibited lower ΔG_bind_ compared to PTX (Table 1) suggesting that the designed PTX analogues may bind tighter to PDEs compared to PTX (Table 1), and could be more effective than PTX in modulating sperm properties which could be conducive for ART. Further, thorough in silico approach, we have designed suicidal inhibitors against PDEs, these terminal inhibitors could be more potent in inhibition of PDE catalytic activity compared to respective reversible inhibitors.

We have chosen PTXm-1 (modified pentoxifylline) for ex vivo studies on sperm to know whether sperm properties modulating ability of PTXm-1 are better than PTX. Data from ex vivo studies have demonstrated that PTXm-1 was able to increase sperm motility, prolong the in vitro sperm survival (Fig. 5), improves the sperm functional competence (Fig. 8a,b and Table 3) and also fertilization potential without affecting the developmental competence of the embryos (Table 4) at fourfold lower concentration compared to PTX. Overall, the beneficial effect of the parent compound (PTX) as well as the PTXm-1 on triggering the motility was similar at early intervals. However, the 24 h in vitro survival was lower in PTX exposed normozoospermic as well as asthenozoospermic spermatozoa. This phenomenon of PTX has been reported in the earlier studies as well^36,37^. Between PTX and PTXm-1, no significant difference was observed with respect to motility enhancement and kinematics except beat cross frequency (BCF), which was significantly higher in PTXm-1 compared to the parent compound (Table 3). Increased tail beating and also lateral head displacement are good indicators of sperm fertilization potential, and these parameters are higher in hyperactivated spermatozoa of fertile men compared to infertile^38^. Further, even though the motility inducing effect on testicular spermatozoa was similar in both PTX and PTXm-1 at the early intervals (up to 1 h), prolonged exposure to PTX resulted in decreased motility. This phenomenon has also been reported earlier in ejaculated spermatozoa, which further emphasizes the superior effect of PTXm-1 over the parent molecule. In addition, our earlier report has shown that both PTX and PTXm-1 do not have any cytotoxic effect on the mouse bone marrow cells. However, PTXm-1 has significantly lower genotoxic effect on mouse bone marrow cells in vivo and human lymphocytes cells in vitro^39^.

One of the major concerns of using PTX in sperm preparation is the premature acrosome reaction in spermatozoa^29–31,36^ which is an undesirable change in the spermatozoa as capacitated spermatozoa has limited survival potential^30,31^. Even in our study, PTX group showed significantly higher acrosome reacted sperm compared to the control and PTXm-1 groups (Fig. 6a), which corroborate the detrimental effect of PTX and the beneficial effects of PTXm-1. Further, significantly higher DNA damage observed in the spermatozoa exposed to the PTX indicates its adverse effects on DNA integrity which agrees with previous report^40^. On the contrary, the chemical modification of PTX significantly reduced the premature acrosome reaction (Fig. 6) and also DNA damage (Fig. 7).

Increase in sperm motility and other physiological changes that are prerequisite for fertilization are dependent on intracellular cAMP level^15,41,42^. Elevation of cAMP level by PTX has been associated with sperm motility enhancement^32,43^, which is confirmed by our result. Further, our results suggest that chemical modification of PTX did not affect the cAMP level (Fig. 8a) and also phosphorylation of tyrosine proteins in spermatozoa (Fig. 8b).

Our results recapitulate previously reported data that PTX is known to impair: fertilization and preimplantation embryo development^43^, and embryonic arrest and also retarded development^44^ (Table 4). Both the PTX and the PTXm-1 have exhibited similar effect on the developmental potential, blastocyst rate, hatching rate and expression of genes regulating pluripotency in embryos (Table 4 and Fig. 9). However, higher incidence of degeneration (data not shown), and significantly higher percentage of TUNEL positive cells observed in blastocysts of PTX group compared to PTXm-1 (Table 4) indicates that the embryos derived from the PTX group are inferior.

## Conclusion

In conclusion, we were successful in structure-assisted design of the PTX analogue, PTXm-1, whose beneficial effect on modulating sperm functions, which are conducive for enhanced fertilization and lesser embryo toxicity, is better than the parent molecule. PTXm-1 performed better than PTX, namely: (A) comparable beneficial effect at much lower concentration than the parent molecule, (B) more effective in extending the longevity of spermatozoa, (C) higher progressively motile spermatozoa as well as higher sperm survival of asthenozoospermic and testicular spermatozoa, (D) slower acrosome reaction, (E) better DNA integrity in spermatozoa, and (F) superior embryo quality. Therefore, PTXm-1 could be a better pharmacological agent than PTX for aiding sperm motility/function enhancement. Further studies are necessary to understand whether the PTXm-1 binds to any other target proteins (other than PDEs) involved in sperm motility enhancement. Future ex vivo studies on the effect of covalent inhibitors and other reversible inhibitors of PDEs on spermatozoa could help to discover more potent sperm function modulators for ART.

## Materials and methods

### Cloning, expression and purification of catalytic domains of PDE4A, PDE4D and PDE10A

Cloning in appropriate vectors, heterologous expression in *Escherichia coli* Rosetta 2 (DE3) competent cells, and subsequent purification of catalytic domains of human *PDE4A*, *PDE4* and *PDE10A* (supplementary materials and methodology).

### Isothermal titration calorimetry binding studies

The equilibrium molar dissociation constant (K_d_), stoichiometry (N) and thermodynamic parameters of the binding of pentoxifylline (PTX) to the catalytic domains of PDE4A, PDE4D and PDE10A were determined with ITC (supplementary materials and methodology).

### Mapping of nucleophilic residues in the substrate binding pocket of PDEs

The properties of the binding pocket of the PDEs were analysed using SiteMap tool in Schrodinger v2019-3 (Schrodinger, LLC). The sequence similarity in PDEs was analysed by performing sequence alignment using Clustal Omega^45^. The residues identified by SiteMap tool was screened for presence of nucleophilic residue. Mapping of nucleophilic residues was done for the binding pockets of PDE4A (PDB ID: 3TVX)^35^, PDE4D (PDB ID: 1ZKN)^46^ and PDE10A (PDB ID: 4HEU)^47^.

### in silico generation of PTX and PTX analogues

Based on the SiteMap analysis of PTX binding pocket of the PDEs, the PTX and the PTX-analogous were generated using 3-D builder tool in Maestro Suit. The ligand structures were further optimized, and energy minimized using OPLS3e force field^48^. Flow chart of analogues design is provided in supplementary material (Supplementary Fig. S11).

### Reversible and covalent docking

Reversible and covalent docking of analogues were carried out on crystal structures of PDE4A (PDB: 3TVX)^35^, PDE4D (PDB: 1ZKN)^46^ and PDE10A (PDB: 4HEU)^47^. MD simulations for these docked complexes were performed using Desmond tool of Schrodinger (supplementary materials and methodology).

### Synthesis of modified pentoxifylline, PTXm-1 (1-[(6E)-7-(6-methoxynaphthyl)-5-oxohept-6-en-1-yl]-3,7-dimethyl-3,7-dihydro-1H-purine-2,6-dione)

Pentoxifylline (0.1 mol) was treated with 6-methoxynaphthaldehyde (0.1 mol) in the presence of sodium hydroxide (10%, 15 mL) and alcohol (l50 mL). The reaction mixture was stirred at room temperature for 4–5 h. The reaction completion was monitored by TLC. Then it was filtered, dried and recrystalised by ethanol and dimethylformamide (DMF) solvents. Further details are given in our earlier report^39^.

### Human semen samples

All the experiments performed were in accordance with NDCT (New Drug and Clinical Trial) rules and ICH-GCP (International Conference on Harmonization- Good Clinical Practice) guidelines for biomedical Resarch on Human participants by ICMR (Indian Council of Medical Research), and the instituional ethical commette guidelines and regulation of KMC, Manipal. The study included subfertile men with normozoospermic and asthenozoospermic semen profile (Table 2) visiting Andrology laboratory, Kasturba Hospital and Medical College, Manipal from September 2015 to September 2018. Study was approved by Institutional Ethics committee of Kasturba Medical College, Manipal Academy of Higher Education, Manipal (IEC 155/205). Written informed consent was taken from the subjects to use their leftover semen samples after the completion of routine semen analysis. For the testicular spermatozoa, testicular tissues from men diagnosed with prostate cancer who were undergoing orchidectomy, were recruited for the study. A prior approval was taken for the study from Institutional committee of Kasturba Hospital, Manipal Academy of Higher Education, Manipal (IEC 409/2020). The tissue was collected from Urology Operation Theater in sterile PBS (details provided in supplementary materials and methodology).

### Drug preparation

PTX purchased from Sigma-Aldrich, USA (Cat. No. P1784) was dissolved in Earl’s balanced salt solution (bicarbonate buffered EBSS, Sigma-Aldrich, Cat. No. E2888) containing 0.1% bovine serum albumin (BSA, Sigma-Aldrich, Cat. No. A3311) to prepare a stock solution of 1 M concentration. For the experiments, working solution (1 mM) was freshly prepared, before each experiment. PTXm-1 stock was made in dimethyl sulfoxide (DMSO) and diluted with EBSS. The concentration of PTX (1 mM)^13,36^ and PTXm-1 (0.25 mM) used in this study was based on our earlier reports^39^.

### Preparation of sperm samples

For the study, subjects with 2–7 days of sexual abstinence were asked to provide their ejaculates by masturbation. After the completion of semen analysis (WHO, 2010), the leftover semen sample from each subject was used for experiment within 1 h of collection and was equally divided into four groups. The semen samples were washed twice with EBSS media (with bicarbonate buffer) by centrifuging at 500 g for 8 min to remove the seminal plasma^49^. The final pellet obtained were overlaid with: (1) EBSS containing 0.1% BSA- (control, C); (2) EBSS containing 0.01% (v/v) of DMSO- (vehicle control, VC); (3) EBSS containing 1 mM of PTX; and (4) EBSS containing 0.25 mM PTXm-1. The EBSS medium used was with bicarbonate buffer. The samples were incubated for 1 h at 37 °C and 5% CO_2_. The overlay containing motile fraction of capacitated spermatozoa were collected carefully without disturbing the pellet and used for further analysis.

### Motility and sperm kinematics

The swim-up fraction containing motile spermatozoa were assessed for motility using manual method and sperm motion characteristics using computer assisted sperm analysis (CASA) as described earlier^50^. For manual motility assessment, ~ 10 µL of sperm suspension was placed on a glass slide and observed under light microscope (400 × magnification). Percentage of spermatozoa with total and progressive motility was calculated. For CASA, 8 µL sperm suspension was placed on a clean microscopic slide, covered with coverslip (18 mm × 18 mm). Motion characteristics such as straight line velocity (VSL), curvilinear velocity (VCL), amplitude of lateral head displacement (ALH), average path velocity (VAP), beat cross frequency (BCF), linearity (LIN), straightness (STR), and balancing (WOB) were assessed in the spermatozoa from asthenozoospermic semen samples under trinoculer microscope (UB200i, 100 × magnification, phase contrast objective) in at least 13 random fields per sample by using ISAS software (Prosier, Spain).

### Testicular spermatozoa

Testicular tissue from men diagnosed with prostate cancer and admitted for orchiectomy (N = 8, age 74.33 ± 5.61 years) were collected from Urology operation theater in sterile PBS. The testis was transferred to Petri dish, washed in phosphate buffered solution (PBS) and cut into small pieces. Seminiferous tubule contents were released into the Petri dish containing EBSS medium with 0.1% BSA by milking the seminiferous tubules with the help of with the help of sterile glass slides. The cell suspension was screened for the presence of testicular spermatozoa under inverted microscope (200 × magnification). The cell suspension was divided into four equal parts- (a) Control (C): EBSS media containing 0.1% BSA; (b) Vehicle control (VC): EBSS containing 0.01% of DMSO; (c) PTX: EBSS containing 1 mM of PTX, and (d) PTXm-1; EBSS containing 0.25 mM PTXm-1. The testicular sperm suspension was processed by discontinuous gradient technique using 40% and 80% density solutions (VGRAD40, VGRAD-80, VITROMED, Germany). The discontinuous gradient column was prepared by adding 1 mL of 80% and 40% gradient solutions sequentially in a sterile test tube. The testicular cell suspension was carefully added above the gradients and centrifuged at 500 g for 20 min. The pellet obtained after centrifugation was further washed by centrifuging at 400 g for 8 min twice. The pellet obtained was resuspended with EBSS media containing DMSO, PTX or PTXm-1 and was transferred to Petri dishes containing 2 mL of respective medium. The testicular cell suspension was incubated at 37 °C and 5% CO_2_ and screened for motility in testicular spermatozoa at 15 min, 1 h and 24 h after incubation.

### Ability of sperm to undergo ionophore-induced acrosome reaction

The ionophore-induced acrosome reaction was assessed using a method described earlier^51^. The swim-up fraction collected (at 1 and 2 h after incubation) from various groups were incubated with calcium ionophore A23187 (Sigma-Aldrich, Cat. No. C7522) at 37 °C for 1 h (N = 28, normozoospermic). Sperm suspension was washed with phosphate buffered solution (PBS), centrifuged at 300 g for 10 min and fixed on a coverslip using methanol. The spermatozoa were stained with Fluorescein isothiocyanate-conjugated *Pisum sativum* agglutinin (FITC-PSA, Sigma-Aldrich, Cat. No. L0770), washed with Milli-Q water, counterstained with propidium iodide (PI) and then mounted on slide using mounting media. Percentage of acrosome reacted spermatozoa (without green florescence in acrosome region) were counted in 500 spermatozoa by observing under fluorescence microscope (400x).

### Assessment of sperm DNA integrity

The sperm DNA damage was evaluated in sperm suspension (N = 28, normozoospermic) by terminal deoxynucleotidyl transferase dUTP Nick End Labelling (TUNEL) assay kit (Rosche diagnosticis, Cat. No. 12156792910) as explained by Isaac et al^52^. Briefly, swim-up fraction containing spermatozoa was collected after 24 h of incubation at 37 °C and 5% CO_2,_  fixed on coverslips using 4% paraformaldehyde (PFA) and washed with PBS. After 1 h of permeabilization using 0.1% Triton X-100, spermatozoa were again washed in PBS. Spermatozoa were then incubated with TUNEL reaction mixture for 1 h in dark, counter stained with DAPI (4’,6-diamidino-2-phynylindole) and percentage of spermatozoa with DNA damage was assessed in minimum of 500 spermatozoa by observing under fluorescence microscope (400x).

### Measurement of intracellular cyclic adenosine monophosphate (cAMP) in spermatozoa

To measure the intracellular cyclic adenosine monophosphate (cAMP) the motile sperm fraction collected at 1 h after incubation from four groups (N = 8, normozoospermic), having at least 3 million spermatozoa, were sonicated for 30 s using a water bath sonicator (Sonics Vibra-Cell VCX130 Ultrasonic, Amplitude 30%). Sperm suspension was centrifuged at 800 g for 10 min and supernatant obtained was frozen at − 80 °C until further analysis. The cAMP level was measured using cAMP test kit as explained by the manufacturers (Elabscience®, Cat. No. E-EL-0056) as described in our earlier report^51^. Briefly, 50 μL of cAMP detection standard or test samples were added to each well of the multi well plate, followed by addition of biotinylated detection solution (50 μL). After 45 min of incubation at 37 °C, the solutions from each well were decanted. The wells of the plate were further washed 3 times using wash buffer and then incubated with 100 μL horse radish peroxidase (HRP) conjugate for 30 min at 37 °C. Further, each well of the multi well plate was washed repeatedly (5 times) and then incubated with 90 μL substrate reagent for 15 min at 37 °C. Finally, the reaction was stopped by adding 50 μL of stop solution and, immediately OD_450nm_ of the each well was measured using Skanit software 5.0 (Multiskan, Photometer, Thermoscientific). The assay was performed in duplicates and standard curve was plotted. The concentration of cAMP was extrapolated from the standard curve.

### Assessment of tyrosine phosphorylation of sperm proteins by immunofluorescence

Assessment of tyrosine phosphorylation of spermatozoa was performed from each group at 1 and 4 h after incubation at 37 °C and 5% CO_2_ which were fixed on coverslip using 4% PFA. Permeabilization of fixed spermatozoa was done by incubating with 0.1% Triton X-100 in PBS for 10 min followed by 2 washes in PBS. The cells were incubated with blocking solution (PBS containing 1% BSA and 0.1% Tween-20) for 1 h followed by incubation at 4 °C overnight with anti phospho-tyrosine antibody (1:300 dilution, Clone PY20, Merck Millipore, Cat. No. 05–947). After washing, the spermatozoa were incubated with anti-mouse IgG-FITC (1:1000, Santa Cruz, USA, Cat. No. S6MQT0101), cells were counterstained with DAPI and observed under fluorescence microscope (1000x, oil immersion). The number of tyrosine phosphoprotein positive spermatozoa were counted in total of 500 spermatozoa and the result was expressed in percentage spermatozoa^34^.

### Effect of PTXm-1 exposure on fertilization and early embryo development: animal experiments

All experiments and animal handing were conducted in accordance with the institutional guidelines and the national guidelines set by committee for control and supervision of experiments on animals (CPCSEA). A prior approval was obtained from Institutional Animal Ethical Committee (IAEC/KMC/78/2015) and animal studies were carried out in observance with ARRIVE guidelines^53^. Inbred female Swiss albino mice (8 weeks) maintained at the Central Animal Research Facility, Kasturba Medical College, Manipal, Manipal Academy of Higher Education, Manipal were used for the experiments. Animals were housed in a continuously controlled environment, with standard conditions of temperature (25 ± 2 °C), humidity (45–55%) and light–dark cycle (12 h of light and 12 h of dark) with standard diet and water ad libitum.

### Superovulation

The adult female *Swiss albino* mice were stimulated using 5 IU of pregnant mare serum gonadotropin (PMSG) and 10 IU of human chorionic gonadotropin (hCG) at an interval of 48 h. At 13 h post hCG administration, the cumulus oocyte complexes (COCs) were collected by teasing the oviduct into M2 medium.

### in vitro fertilization and embryo development

To assess the effect of PTXm-1 on fertilization outcome and the early embryo development, Swiss albino mice were used. The male mice (6 to 8 weeks, N = 6 per trial, total 3 trials) were humanely sacrificed by cervical dislocation and cauda was dissected out. The IVF was performed as described in our earlier report^39^. Briefly, the cauda was teased in a Petri dish containing EBSS (with 0.1% BSA) culture media and incubated at 37 °C, 5% CO_2_ incubator. After 2 h of incubation, the spermatozoa were collected and divided equally into 4 parts as C, VC, PTF and PTXm-1 groups. The sperm suspension was centrifuged at 250 g for 8 min and the supernatant was discarded. The final pellet obtained were carefully overlaid with: (1) EBSS media containing 0.1% BSA- (C); (2) EBSS containing 0.01% (v/v) of DMSO (VC); (3) EBSS containing 1 mM of PTX; and (4) EBSS containing 0.25 mM PTXm-1. The spermatozoa were then incubated for 1 h at 37 °C and 5% CO_2_ incubator for the motile sperm to swim up. The overlay containing motile sperm fraction of were collected carefully without disturbing the pellet, and were used for preparation of insemination droplets (80 µL) which was covered with paraffin oil (Cat. No. 61822605001730, Merck, Germany). The female mice were sacrificed humanely by cervical dislocation (N = 12 per trial, total 3 trials). COCs collected from the superovulated female mice were randomly divided into 4 groups and were transferred to insemination droplets of C, VC, PTX and PTXm-1. Fertilization was assessed at 12 h post-insemination and normally fertilized oocytes (oocytes with 2 PN and 2 PB) were cultured further in M16 media till the blastocyst stage. Blastocyst rate, hatching rate and, DNA damage and total cell number was assessed in blastocysts^54^.

### RNA extraction, cDNA synthesis and real-time quantitative polymerase chain reaction for pluripotency markers

RNA was extracted from blastocyst stage embryos (30 per group) using an RNA isolation aqueous micro kit (Catalogue No. AM1931; Ambion, Foster City, CA, USA) as explained^54^. Briefly, embryos were lysed using lysis buffer to release total RNA and was eluted using elution buffer. RNA obtained was treated with DNase for 20 min at 37 °C and stored at − 80 °C. cDNA was synthesized using a Superscript first strand cDNA synthesis kit for reverse transcription polymerase chain reaction (Catalogue No. E6300; NewEngland Biolabs, Ipswich, MA, USA).

Quantitative PCR for relative expression of Sox2, Nanog, and Oct4 mRNA was done using StepOne real-time PCR system (Applied Biosystems, California, USA) with TaqMan® Gene Expression Assay probes and TaqMan® Fast Advanced MixThe relative mRNA expression of Cdx2 was also estimated using real-time PCR system with SYBER Green chemistry (TaKaRa, Shiga, Japan, Cat. No. RR420A). All the data were normalized with GAPDH housekeeping gene as an internal control. The relative expression levels were calculated in terms of fold change. The IDs for TaqMan® Gene Expression Assay probes used in this study and the specific primers used for Cdx2 gene and GAPDH housekeeping gene are given in Supplementary Table S1.

### Statistical analysis

All the data are presented as mean and standard error (mean ± SEM), except embryo developmental parameters which are represented as percentage data. The statistical significance level of mean ± SEM was calculated using one-way analysis of variance (ANOVA) followed by post-hoc Turkey test while the percentage data was analyzed by Chi square test using GraphPad InStat 3.0 statistical package (GraphPad Inc., USA). *P* value less than 0.05 was considered as statistically significant. All the graphs were plotted using Origin 6.0 (Origin Lab Corporation, Northampton, MA, USA).

## Supplementary Information

Supplementary Information.

## Abbreviations

AC: Adenylyl cyclase
AR: Acrosome reaction
ART: Assisted reproductive technologies
cAMP: Cyclic adenosine monophosphate
ΔG_bind_: Free energy of binding
ICSI: Intracytoplasmic sperm injection
ITC: Isothermal titration calorimetry
IUI: Intrauterine insemination
IVF: In vitro fertilization
MD: Molecular dynamics
PDEs: Phosphodiesterases
PTX: Pentoxifylline
PDEIs: Phosphodiesterase inhibitors
K_d_: Equilibrium molar dissociation constant
