# Supplementary material for: Structure-based redesigning of pentoxifylline analogs against selective phosphodiesterases to modulate sperm functional competence for assisted reproductive technologies
Source: Sci Rep. 2021 Jun 10;11:12293. doi: 10.1038/s41598-021-91636-y (PMC8192908; doi:10.1038/s41598-021-91636-y)
Supplement: Supplementary file 1 — Supplementary Information. [file 41598_2021_91636_MOESM1_ESM.pdf]

Supplementary Information

Structure-based redesigning of pentoxifylline analogs against selective phosphodiesterases to modulate sperm functional competence for assisted reproductive technologies

Mutyala Satish[1,#], Sandhya Kumari[2,#], Waghela Deeksha[1], Suman Abhishek[1], Kulhar Nitin[1], Satish Kumar Adiga[2], Padmaraj Hegde[3], Jagadeesh Prasad Dasappa[4], Guruprasad Kalthur[2,*], Eerappa Rajakumara[1,*]

[1]Macromolecular Structural Biology Lab, Department of Biotechnology, Indian Institute of Technology Hyderabad, Kandi, Sangareddy, Telangana 502285, India

[2]Department of Clinical Embryology, Kasturba Medical College, Manipal, Manipal Academy of Higher Education, Manipal, Karnataka, 576104, India.

[3]Department of Urology, Kasturba Medical College, Manipal, Manipal Academy of Higher Education, Manipal, Karnataka, 576104, India.

[4]Department of Chemistry, Mangalore University, Mangalagangothri, 574199, Karnataka, India

[#]These authors contributed equally to this manuscript

Corresponding Authors:

[*]ER. E-mail: eraj@bt.iith.ac.in; [*]GK. E-mail: guru.kalthur@manipal.edu

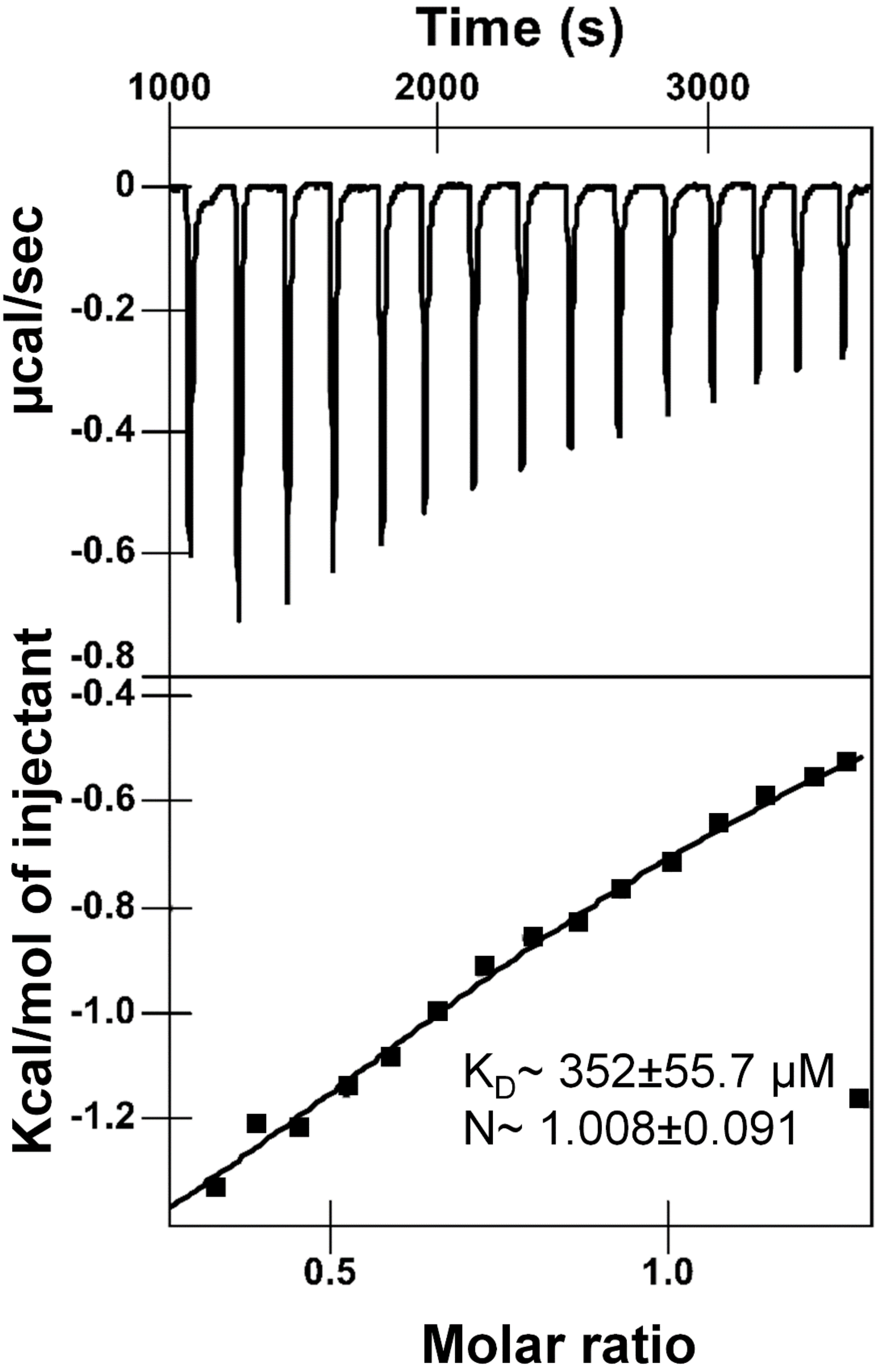

Supplementary Figure S1. Competitive binding studies between PTX with PDE10A -PTXm-1 complex. Enthalpy plots for the binding of PTX to PDE10A and PTXm-1

complex. The inset lists the measured molar dissociation constant (K_d), stoichiometry (N) and change in an enthalpy (ΔH) and an entropy (ΔS).

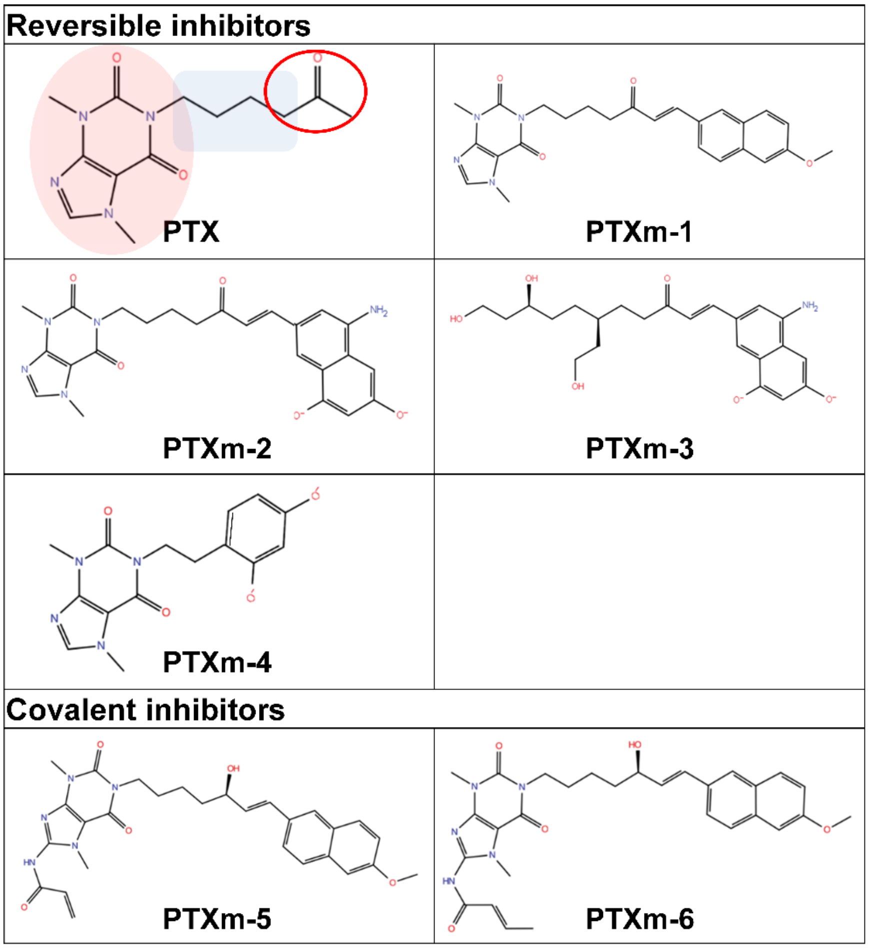

Supplementary Figure S2. 2D structures of pentoxifylline and designed pentoxifylline derivatives. The purine ring, the aliphatic chain and the ketone tail are shown in red shade, blue shade and red circle, respectively.

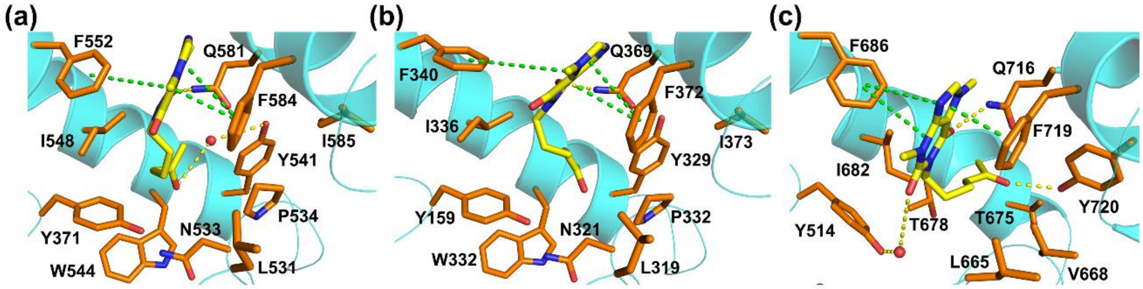

Supplementary Figure S3. Interaction of pentoxifylline (PTX) with PDEs at the end of MD simulations. PTX complexed with (a) PDE4A, (b) PDE4D and (c) PDE10A. PTX is shown in yellow color. Water is shown as red sphere. H-bond and π-stacking interactions are shown as yellow and green dashes, respectively.

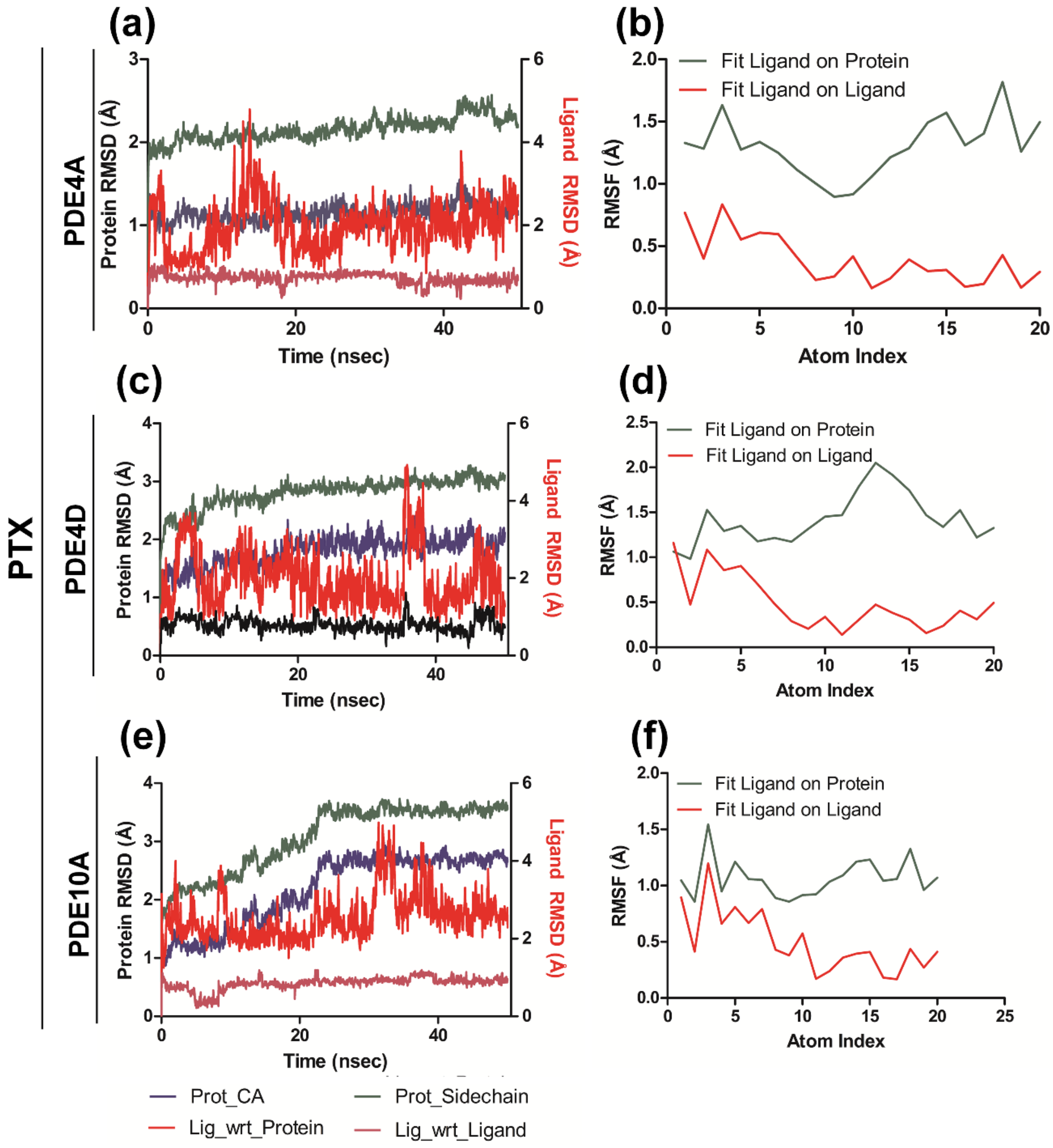

Supplementary Figure S4. RMSD of PDEs-PTX complex and RMSF profiles of PTX in the PDEs binding pocket during MD simulation. (a) RMSD of PDE4A and PTX. (b) RMSF of PTX in the binding pocket of PDE4A. (c) RMSD of PDE4D and PTX. (d) RMSF of PTX in the binding pocket of PDE4D. (e) RMSD of PDE10A and PTX. (f) RMSF of PTX in the binding pocket of PDE10A.

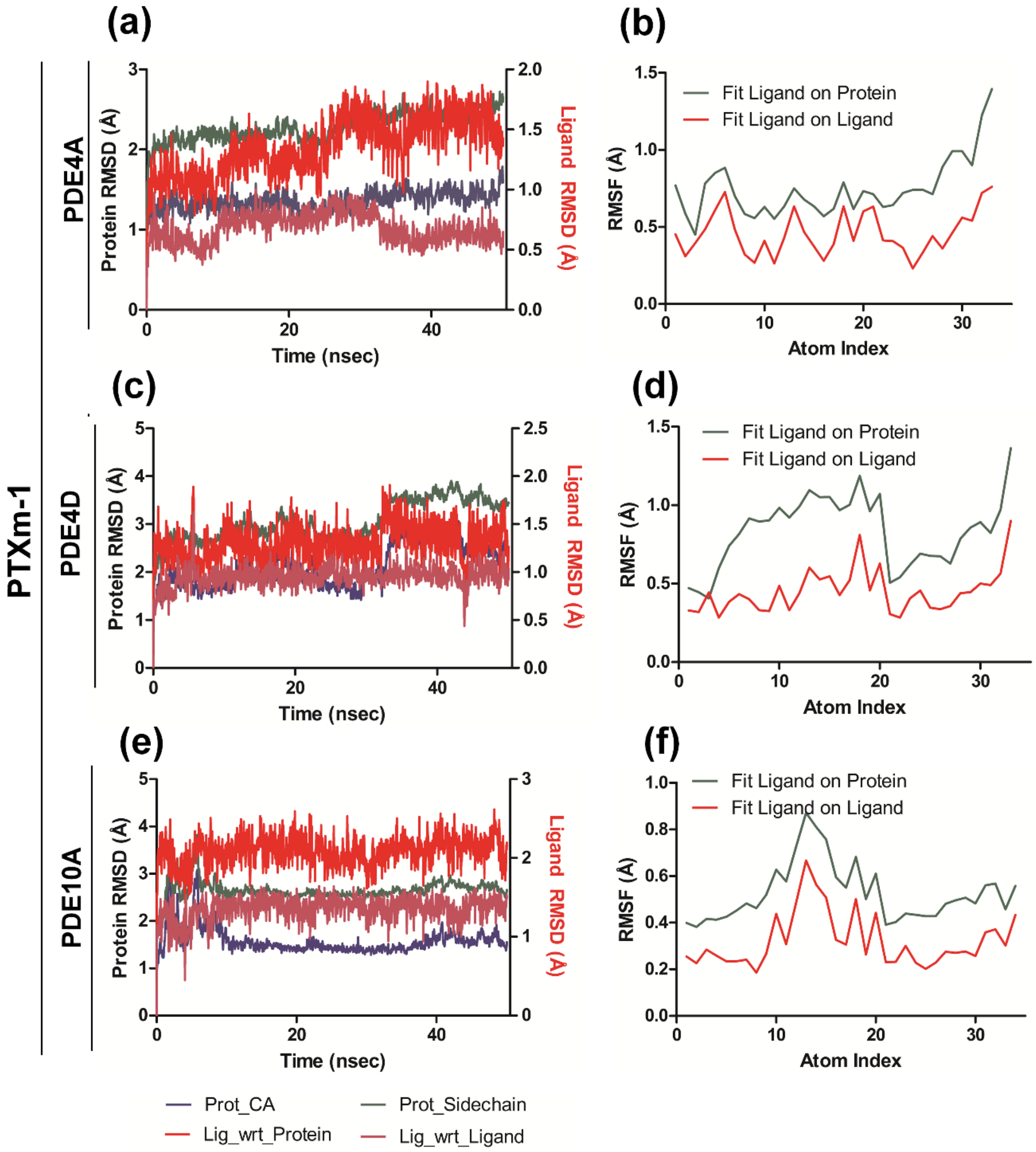

Supplementary Figure S5. RMSD of PDEs-PTXm-1 complex and RMSF profiles of PTXm-1 in the PDEs binding pocket during MD simulation. (a) RMSD of PDE4A and PTXm-1. (b) RMSF of PTXm-1 in the binding pocket of PDE4A. (c) RMSD of PDE4D and PTXm-1. (d) RMSF of PTXm-1 in the binding pocket of PDE4D. (e) RMSD of PDE10A and PTXm-1. (f) RMSF of PTXm-1 in the binding pocket of PDE10A.

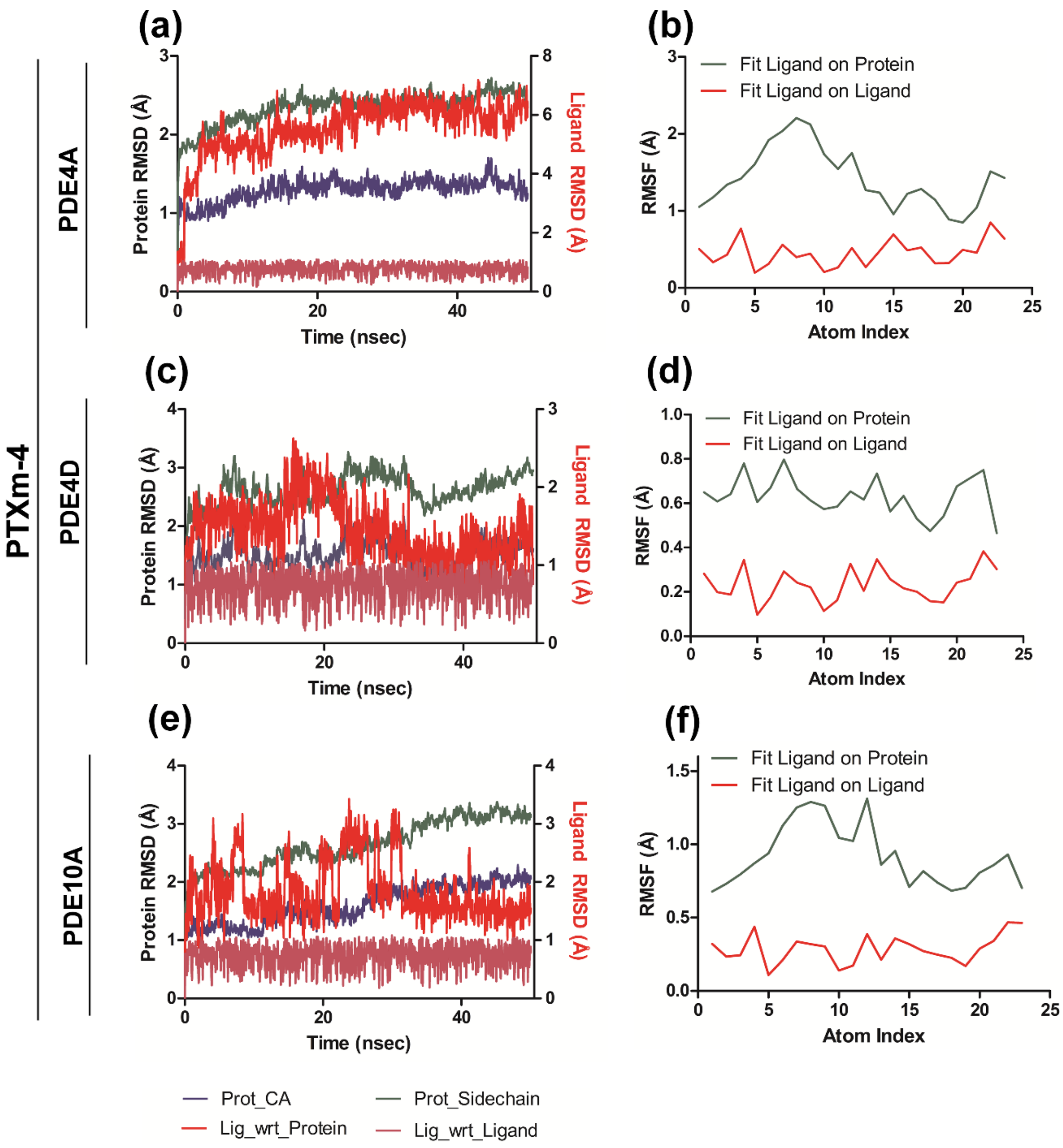

Supplementary Figure S6. RMSD of PDEs-PTXm-4 complex and RMSF profiles of PTXm-4 in the PDEs binding pocket during MD simulation. (a) RMSD of PDE4A and PTXm-4. (b) RMSF of PTXm-4 in the binding pocket of PDE4A. (c) RMSD of PDE4D and PTXm-4. (d) RMSF of PTXm-4 in the binding pocket of PDE4D. (e) RMSD of PDE10A and PTXm-4. (f) RMSF of PTXm-4 in the binding pocket of PDE10A.

(a)

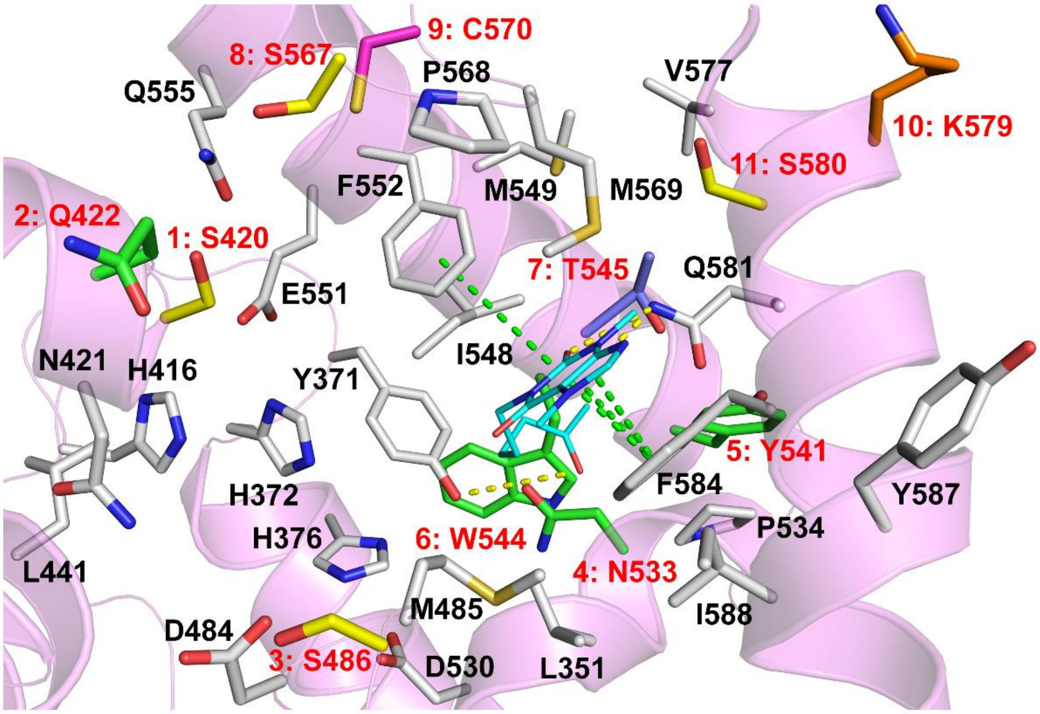

(b)

Sl.	Protein	Relative position of residues										
		1	2	3	4	5	6	7	8	9	10	11
1.	PDE4A	S	Q	S	N	Y	W	T	S	C	K	S
2.	PDE4D	S	Q	S	N	Y	W	T	S	C	K	S
3.	PDE10A	S	S	A	S	T	T	A	I	M	Q	G

Supplementary Figure S7. Mapping of nucleophilic residues in the PDEs. (a) Structure of PDE4A-PTX complex (PDB ID: 3TVX) showing nucleophilic residues Serine (Yellow), Cysteine (Magenta), Lysine (Orange) and Threonine (Blue) along with other binding pocket residues (grey). The non-nucleophilic residues in green indicate that the position is occupied by nucleophilic residues in PDE10A. (b) Table showing the residues at the relative position of nucleophilic residues (marked in a) in the binding pocket of PDE4A, PDE4D and PDE10A.

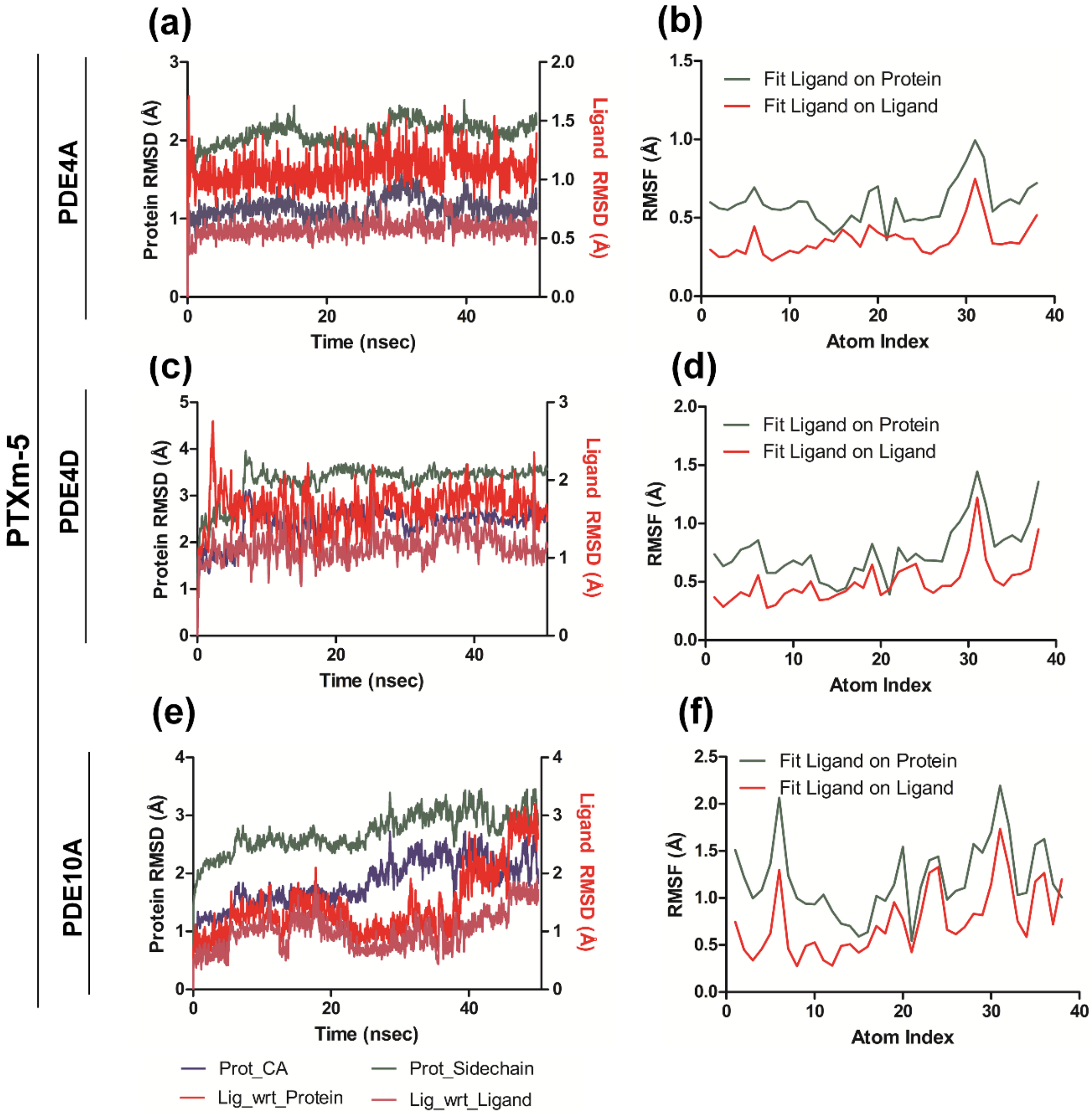

Supplementary Figure S8. RMSD of PTXm-5 reversibly bound to PDEs and RMSF profiles of PTXm-5 in the PDEs binding pocket during MD simulation. (a) RMSD of PDE4A and PTXm-5. (b) RMSF of PTXm-5 in the binding pocket of PDE4A. (c) RMSD of PDE4D and PTXm-5, (d) RMSF of PTXm-5 in the binding pocket of PDE4D. (e) RMSD of PDE10A and PTXm-5. (f) RMSF of PTXm-5 in the binding pocket of PDE10A.

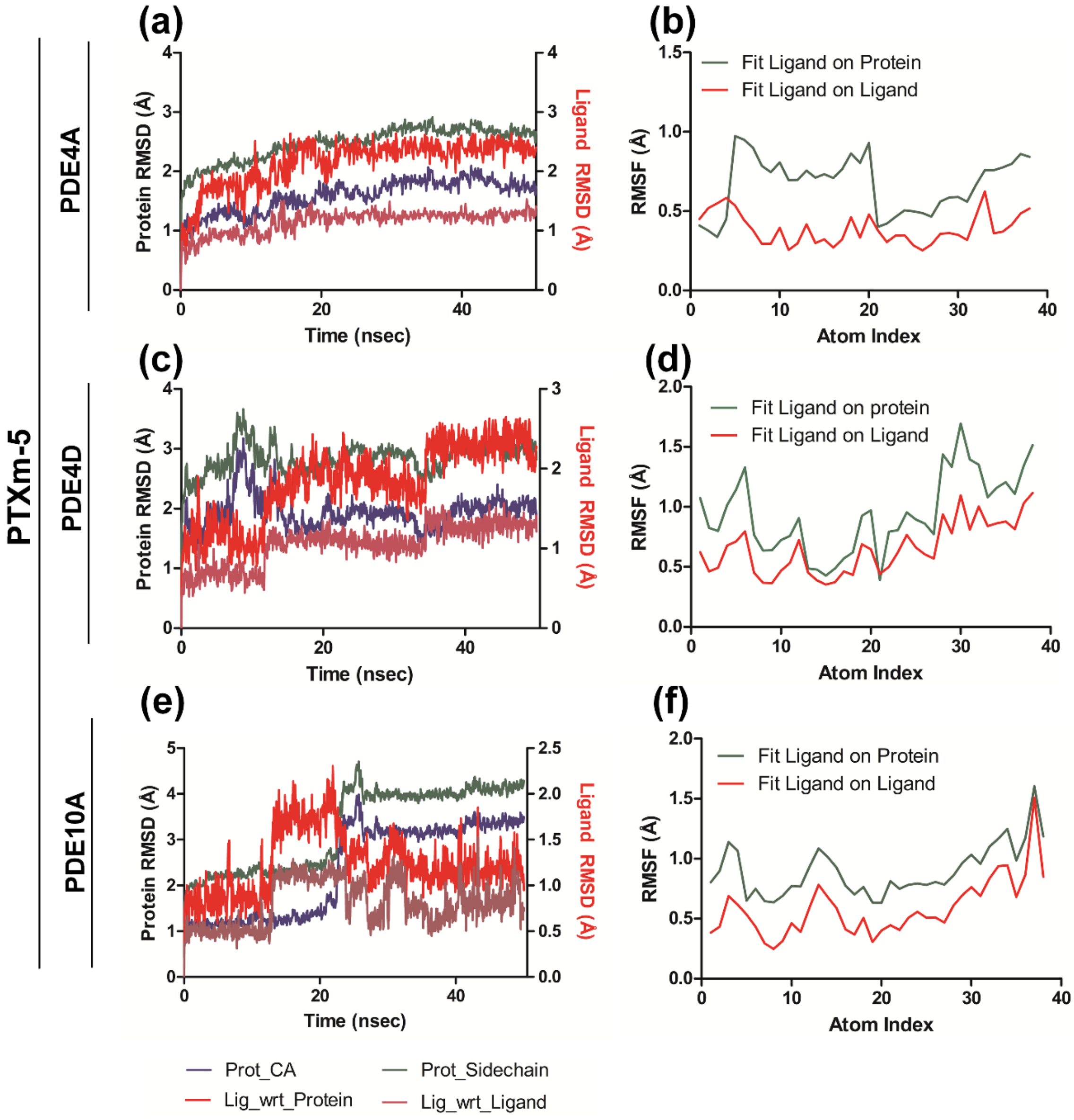

Supplementary Figure S9. RMSD of PTXm-5 covalently bound to PDEs and RMSF profiles of PTXm-5 in the PDEs binding pocket during MD simulation. (a) RMSD of PDE4A and PTXm-5. (b) RMSF of PTXm-5 in the binding pocket of PDE4A. (c) RMSD of PDE4D and PTXm-5. (d) RMSF of PTXm-5 in the binding pocket of PDE4D. (e) RMSD of PDE10A and PTXm-5. (f) RMSF of PTXm-5 in the binding pocket of PDE10A.

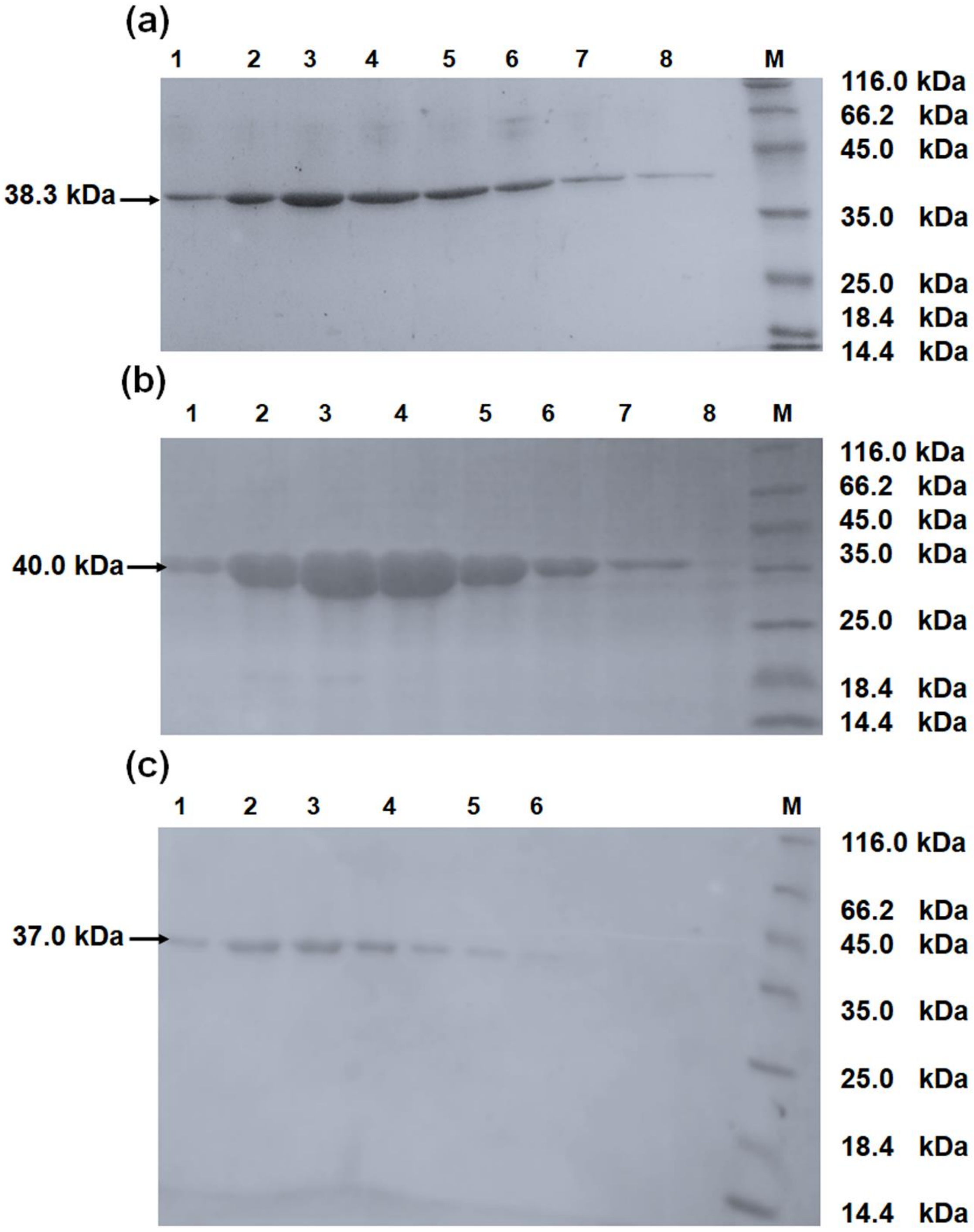

Supplementary Figure S10. Analysis of the purity of the size exclusion chromatography purified catalytic domain of PDEs on 12% SDS-

PAGE. (a) PDE4A (b) PDE4D (c) PDE10A. Lanes are numbered with eluted fraction collection. M stands for protein marker. Molecular weight of the PDEs are labelled on left side of gel.

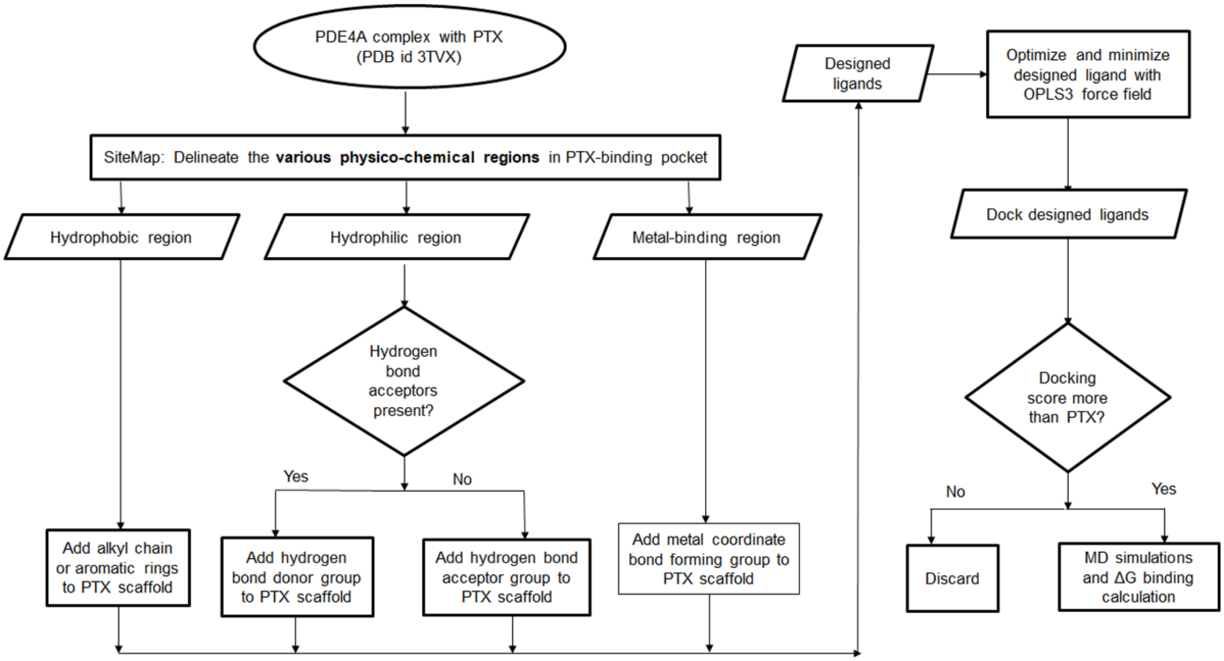

Supplementary Figure S11. PTX analogues design flow chart

Supplementary Table S1. List of primers used in the study. (a) Primer list for real time PCR using SyBr-Green chemistry. (b) Primer list for real time PCR using Taqman chemistry

(a)

Gene	Forward primer	Reverse primer
GAPDH	AGGTCGGTGTGAACGGATTTG	TGTAGACCATGTAGTTGAGGTCA
Cdx2	CAAGGACGTGAGCATGTATCC	GTAACCACCGTAGTCCGGGTA

(b)

Gene	Forward primer
GAPDH	Mm99999915_g1
Oct4	Mm03053917_g1

Sox2	Mm03053810_s1
Nanog	Mm02019550_s1

Supplementary Materials and Methods

Cloning, expression and purification of catalytic domains of PDE4A, PDE4D and PDE10A

The full length human *PDE4A* (1005 bp), *PDE4D* (981 bp) and *PDE10A* (957 bp) cDNAs, cloned in pET28a(+) expression vector, were purchased from Biomatik, Canada. Catalytic domain of each protein was cloned between BamHI and XhoI restriction sites in 6x-His-SUMO-tagged bacterial expression vector. The catalytic domain constructs of PDE4A, PDE4D and PDE10A were transformed in *E. coli* Rosetta 2 (DE3) competent cells for expression. After growing the transformed cells at 37 °C, till the OD_{600nm} reached 0.5-0.7, the culture containing constructs of PDE4A and PDE4D were induced with 0.1 mM IPTG and the cultures were incubated at 15 °C for 16-18 h, whereas those with PDE10A constructs were induced with 0.5 mM at incubated for 35-40 h followed by cell harvesting. Harvested cells were suspended in lysis buffer (500 mM NaCl, 50 mM Tris-Cl pH 8.0, 10 mM Imidazole, 3 mM β-mercaptoethanol) and lysed by sonication (Qsonica). The lysates were cleared by centrifugation at 20,000g for 1h and supernatant was passed through Ni-NTA affinity column (His-Trap, GE Healthcare). Eluted proteins were subjected to salt exchange with a buffer containing 100 mM NaCl, 50 mM Tris-Cl pH 8.0, 3 mM β-mercaptoethanol. The collected proteins were further purified using ion-exchange chromatography (Q column, Hi-Trap, GE Healthcare) followed by overnight treatment with ULP1 at 4 °C to cleave 6xHis-SUMO tag. The tag was separated from proteins by a subsequent Ni-NTA purification. The proteins were further purified on size exclusion 16/600 200 pg column (GE Healthcare) pre-equilibrated with a buffer (100 mM

NaCl, 50 mM Tris pH 8.0, 5% Glycerol, 3 mM β-mercaptoethanol). After purifying with a gel filtration column, purity was determined on 12% SDS-PAGE and all three proteins were more than 95% pure (Supplementary Figure S10). Purified proteins were concentrated to 20mg/ml, flash cooled with liquid nitrogen and stored at -80°C.

Isothermal titration calorimetry (ITC) binding studies

The equilibrium molar dissociation constant (K_d), stoichiometry (N) and thermodynamic parameters of the binding of pentoxifylline (PTX) to the catalytic domains of PDE4A, PDE4D and PDE10A were determined at 25°C, using Affinity ITC (TA instruments). Protein and ligand solutions for titration were prepared in the same buffer. The reference cell was filled with a distilled water and the sample cell was filled with 350 µL of either PDE4A or PDE4D or PDE10A (75-500 µM). PTX (5-10 mM) as titrant was sequentially injected in 2 µL per injection (for a total 20 injections), at a regular interval of 120-150s, while stirring at 125 rpm. The ITC generated data were processed using NanoAnalyze software (TA Instruments). The ITC data was deconvoluted with the “independent” curve fitting model using a nonlinear least-squares algorithm. Binding enthalpy change (ΔH), association constant (K_a), and binding stoichiometry (N) were permitted to vary during the least-squares minimization process. The errors denote standard deviations at 95% confidence interval.

Reversible and covalent docking

Reversible docking was carried out on crystal structures of PDE4A (PDB: 3TVX)[1], PDE4D (PDB: 1ZKN)[2] and PDE10A (PDB: 4HEU)[3]. Before docking, the protein was prepared using ‘Protein Preparation Wizard’ in Maestro interface of Schrödinger v2019-3

(Schrödinger, LLC). During this, the bond order was assigned, hydrogen atoms were added, protonation was carried out using ProPKA[4], H-bonds were optimized, and the heavy atoms were minimized using OPLS3e force field.

The centroid of the SiteMap identified residues was used for grid-generation. The grid was used for performing rigid reversible docking, where the receptor was kept rigid and the ligand was allowed constrained flexibility. The generated PTX and PTX analogous were docked with XP precision docking mode of Glide module of Schrodinger v2019-3 (Schrodinger, LLC). The best poses were selected using the glide score. Negative scores indicate tighter binding.

Covalent docking was carried out using CovDock mode of Glide module. To perform covalent docking, the nucleophilic residues, i.e. C570 of PDE4A, C358 of PDE4D and S563 of PDE10A were targeted for Michael addition reaction with the warhead group in PTXm-5. The centroid of the SiteMap identified residues were used for grid generation to specify the target site of ligand binding. The best binding pose of the ligand was selected based on the Molecular mechanics – Generalized Borne Surface Area (MM-GBSA) scoring, calculated post docking.

The best outputs of all the dockings (reversible and covalent) were subjected to molecular dynamics (MD) simulations to analyze the stability of the ligand in the binding pocket of PDEs.

Molecular dynamics (MD) simulation

MD simulations, for PDE4A, PDE4D and PDE10A reversibly docked with PTX and PTX analogous as well as covalently docked PTXm-5, were undertaken using the

Desmond engine (Desmond Molecular Dynamics System, Version 2.2, D.E. Shaw Research, New York, NY, 2009). The complexes were enclosed in an orthorhombic box (a=b=c=10 Å and α=β=γ=90°), neutralized with calculated counter ions (Na^+ or Cl^-) and solvated with TIP4Pew water model containing 0.15 M NaCl. The systems were minimized and equilibrated with default protocols of the Desmond. The dynamics of the system was calculated with the OPLS3e force field. The long-range electrostatic interactions were calculated using particle-mesh Ewald method[5]. A cut-off radius of 9.0 Å was applied for short-range van der Waals and Coulomb interactions. The systems were simulated under an isothermal-isobaric (NPT) ensemble at 300 K temperature and 1 atm pressure. The temperature and the pressure of the system were maintained using Nose-Hoover thermostat[6] and Martyna–Tobias–Klein methods[7], respectively. An integral time-step of 2 fs was used for the overall simulation. Finally, a 50 ns production MD simulation was performed independently for all the systems.

Reference

1. Recht, M. I. *et al.* Fragment-based screening for inhibitors of PDE4A using enthalpy arrays and X-ray crystallography. *J. Biomol. Screen.* **17**, 469–480 (2012).
2. Huai, Q., Liu, Y., Francis, S. H., Corbin, J. D. & Ke, H. Crystal structures of phosphodiesterases 4 and 5 in complex with inhibitor 3-isobutyl-1-methylxanthine suggest a conformation determinant of inhibitor selectivity. *J. Biol. Chem.* **279**, 13095–13101 (2004).

3. Rzasa, R. M. *et al.* Discovery of selective biaryl ethers as PDE10A inhibitors: Improvement in potency and mitigation of Pgp-mediated efflux. *Bioorganic & Medicinal Chemistry Letters* **22**, 7371–7375 (2012).
4. Li, H., Robertson, A. D. & Jensen, J. H. Very fast empirical prediction and rationalization of protein pKa values. *Proteins* **61**, 704–721 (2005).
5. Darden, T., York, D. & Pedersen, L. Particle mesh Ewald: An N·log(N) method for Ewald sums in large systems. *J. Chem. Phys.* **98**, 10089–10092 (1993).
6. Hoover, null. Canonical dynamics: Equilibrium phase-space distributions. *Phys. Rev. Gen. Phys.* **31**, 1695–1697 (1985).
7. Martyna, G. J., Tobias, D. J. & Klein, M. L. Constant pressure molecular dynamics algorithms. *J. Chem. Phys.* **101**, 4177–4189 (1994).
